# Organ-level gene-regulatory networks inferred from transcriptomic data reveal context-specific regulation and highlight novel regulators of ripening and ABA-mediated responses in tomato

**DOI:** 10.1016/j.xplc.2025.101499

**Published:** 2025-09-03

**Authors:** José D. Fernández, David Navarro-Payá, Antonio Santiago, Ariel Cerda, Jonathan Canan, Sebastián Contreras-Riquelme, Tomás C. Moyano, Diego Landaeta-Sepúlveda, Lorena Melet, Javier Canales, Nathan R. Johnson, José M. Álvarez, José Tomás Matus, Elena A. Vidal

**Affiliations:** 1 Centro de Genómica y Bioinformática, Universidad Mayor, Santiago 8580745, Chile; 2 Agencia Nacional de Investigación y Desarrollo-Millennium Science Initiative Program-Millennium Institute for Integrative Biology (iBio), Santiago 8331150, Chile; 3 Agencia Nacional de Investigación y Desarrollo-Millennium Nucleus in Data Science for Plant Resilience (Phytolearning), Santiago 8370186, Chile; 4 Institute for Integrative Systems Biology (I^2^SysBio), Universitat de València - CSIC, Paterna, 46908 Valencia, Spain; 5 Instituto de Ciencias de la Ingeniería, Universidad de O’Higgins, Rancagua 2820000, Chile; 6 Instituto de Bioquímica y Microbiología, Facultad de Ciencias, Universidad Austral de Chile, Valdivia 5110566, Chile; 7 Centro de Biotecnología Vegetal, Facultad de Ciencias de la Vida, Universidad Andrés Bello, Santiago 8370186, Chile

**Keywords:** gene-regulatory network, *Solanum lycopersicum*, transcriptional regulation, drought, ripening

## Abstract

Tomato (*Solanum lycopersicum*) is a globally important crop, yet the gene-regulatory networks (GRNs) that control its gene expression remain poorly understood. In this study, we constructed GRNs for roots, leaves, flowers, fruits, and seeds by inferring transcription factor (TF)–target interactions from over 10 000 RNA-sequencing libraries using the GENIE3 algorithm. We refined these networks using gene co-expression data and computational predictions of TF binding sites. Our networks confirmed key regulators in important processes, including TOMATO AGAMOUS LIKE 1 and RIPENING INHIBITOR in fruit ripening, and *Sl*ABF2, *Sl*ABF3, and *Sl*ABF5 in abscisic acid (ABA) response in leaves. In addition, we identified novel candidate regulators, including AUXIN RESPONSE FACTOR 2A and ETHYLENE RESPONSE FACTOR E2 in fruit ripening and G-BOX BINDING FACTOR 3 (*Sl*GBF3) in ABA-related and drought pathways. To further validate the GRNs, we performed DNA affinity purification sequencing for *Sl*GBF3 and confirmed the accuracy of our GRN predictions. This study provides a valuable resource for dissecting transcriptional regulation in tomato, with potential applications in crop improvement. The GRNs are publicly accessible through a user-friendly web platform at https://plantaeviz.tomsbiolab.com/tomviz.

## Teaser

This study presents organ-level gene-regulatory networks for tomato, constructed from over 10 000 RNA-sequencing libraries and refined using co-expression data and transcription factor binding predictions. The networks successfully recaptured known regulators of ripening and ABA responses and identified novel candidates, including *Sl*GBF3, which was experimentally validated through DNA affinity purification sequencing. This resource offers new insight into transcriptional regulation in tomato and is publicly accessible online.

## Introduction

Tomato (*Solanum lycopersicum* L.) is one of the world’s most widely cultivated and consumed crops, serving as a model organism for studies of fleshy fruit development, ripening, and plant defense responses (Kimura and Sinha, 2008; Gascuel et al., 2017). Despite its significance, the gene-regulatory networks (GRNs) that control tomato responses to internal and external signals remain largely understudied.

Various approaches exist to help with the identification of transcription factor (TF)–target interactions and generate GRNs. Chromatin immunoprecipitation followed by sequencing (ChIP-seq) and DNA affinity purification sequencing (DAP-seq) can be used to assess TF binding to genomic regions. ChIP-seq provides *in vivo* binding information but is technically demanding owing to the need for high-quality antibodies and optimized protocols for native chromatin extraction (Park, 2009). By contrast, DAP-seq enables genome-wide mapping of TF–DNA interactions using *in vitro*-expressed TFs and genomic DNA, making it particularly suitable for crop species such as tomato. Although both techniques provide valuable insights into TF function, only a limited number of TFs have been studied using ChIP-seq (Fujisawa et al., 2011; Ricardi et al., 2014; Du et al., 2017; Lü et al., 2018; Gao et al., 2019; Lira et al., 2020; Liu et al., 2020; Ding et al., 2022; Tu et al., 2022; Yang et al., 2022; Jiang et al., 2023) or DAP-seq (López-Vidriero et al., 2021; Chong et al., 2022; Huang et al., 2023; Zhu et al., 2023). Alternative approaches to derive binding of multiple TFs at a genome scale on the basis of accessible chromatin sites—such as assay for transposase-accessible chromatin with sequencing (ATAC-seq) and DNase I hypersensitive site sequencing (DNase-seq) in tomato are also scarce and limited in scope. Most ATAC-seq analyses have focused on fruit responses to abiotic stress (Maher et al., 2018; Reynoso et al., 2019; Hendelman et al., 2021; Kajala et al., 2021), whereas DNase-seq studies have focused primarily on fruit development (Qiu et al., 2016; Lü et al., 2018).

Existing biological network models in tomato are focused primarily on protein–protein interactions or gene co-expression networks (GCNs), often derived from small datasets or limited to specific conditions (Ozaki et al., 2010; Fukushima et al., 2012; Gao et al., 2013; Koenig et al., 2013; Pan et al., 2013; Ichihashi et al., 2014; Arhondakis et al., 2016; Kim et al., 2017; Zouine et al., 2017; Xie et al., 2019; Bae et al., 2021; Bizouerne et al., 2021; Kusano et al., 2022; Wang et al., 2023a; Li et al., 2024). Some studies have integrated larger-scale transcriptomic datasets from microarrays and RNA sequencing (RNA-seq) to generate GCNs. Fukushima et al. (2012) analyzed 307 microarrays from 17 experiments, encompassing diverse conditions in a single GCN that was separated into organ-level submatrices. Similarly, Kim et al. (2017) compiled 1473 expression samples from 12 microarray and mRNA-seq studies to generate organ-independent co-expression networks, and Zouine et al. (2017) integrated 29 RNA-seq studies to generate a global GCN. However, these networks are limited in several important respects. They tend to represent only fruit or to combine data from all organs, overlooking organ-level information (Fukushima et al., 2012; Kim et al., 2017; Zouine et al., 2017). They are also based on older tomato genome assemblies (SL2.5 or SL3.0) compared with the current SL4.0. Moreover, GCN approaches are unable to define directed regulatory interactions (Babu et al., 2004; Chai et al., 2014; Swift and Coruzzi, 2017).

GRNs offer a more comprehensive framework for studying TF–target interactions at a genome-wide scale. GRNs infer directed regulatory interactions, identifying key regulatory hubs that orchestrate biological processes (Doidy et al., 2016; Vidal et al., 2020; Escorcia-Rodríguez et al., 2023). In a recent study, we used GRN modeling to investigate TFs involved in sulfate deficiency responses in tomato and other crops (Fernández et al., 2024). However, the absence of comprehensive tomato GRNs that incorporate the most recent gene annotations was a major limitation in our study. Therefore, developing genome-wide GRN models that integrate updated genome assemblies, diverse transcriptomic datasets, and organ-level regulation is crucial for uncovering transcriptional regulatory mechanisms in tomato.

Machine-learning-based approaches such as GENIE3 (Gene Network Inference with Ensemble of Trees) have been widely used to infer GRNs from large-scale transcriptomic datasets (Huynh-Thu et al., 2010). GENIE3 uses an ensemble of regression trees to predict TF–target interactions and has demonstrated high performance in the DREAM4 and DREAM5 network inference challenges (Huynh-Thu et al., 2010; Huynh-Thu and Geurts, 2019). This approach has been successfully used to generate GRNs in various plant species, including *Arabidopsis thaliana*, wheat, and maize (Huang et al., 2018; Harrington et al., 2020; Tu et al., 2020; De Clercq et al., 2021; Chen et al., 2023; Ranjan et al., 2024).

In this study, we integrated large-scale tomato omics datasets to generate, validate, and refine organ-level GRNs. Using an extensive set of transcriptomic data and the GENIE3 algorithm, we generated five reference GRNs for different tomato organs (roots, leaves, flowers, fruits, and seeds). These networks were further enhanced with TF–target interactions derived from accessible chromatin data, predicted TF-binding events, and genome-wide GCNs. The resulting organ-level GRNs provide a robust foundation for exploring diverse biological contexts, from developmental processes to stress responses, offering a valuable resource for addressing unresolved questions in tomato biology. The organ-level GRNs are available at https://plantaeviz.tomsbiolab.com/tomviz.

## Results

### Update of tomato gene models and functional annotations

The most recent genome data available in the SolGenomics Network database (Fernandez-Pozo et al., 2015) is the SL4.0 genome assembly (Hosmani et al., 2019), together with the ITAG4.1 annotation released in January 2020. Although we initially aimed to use ITAG4.1, alignment of RNA-seq libraries revealed the loss of 3393 gene models compared with the previous ITAG4.0 version, including key functional genes such as *RIPENING-INSENSITIVE* (*SlRIN*). To prevent the omission of relevant genes, we integrated the ITAG4.0 and ITAG4.2 beta gene models to develop a new annotation, ITAG4.2-merged, that contained 37 467 genes (Supplemental Tables 1 and 2). Because only 15.53% of tomato genes had a functional annotation in ITAG4.0, we generated an updated annotation for protein-coding genes by integrating diverse sources of evidence. These included Gene Ontology (GO) terms assigned by EggNOG-mapper (Cantalapiedra et al., 2021) and InterProScan (Jones et al., 2014), as well as gene functional annotations compiled in PLAZA 5.0 for tomato (Van Bel et al., 2022). We achieved a functional annotation coverage of 65% for 24 356 out of 37 467 protein-coding genes in ITAG4.2-merged assigned to at least one GO term. This dataset included 9568 unique GO terms, providing biological function annotations for 21 303 genes and molecular function annotations for 23 809 genes, for a total of 982 370 annotations (Supplemental Tables 1 and 3).

To generate an updated list of TFs, we retrieved TF catalogs from various repositories, and only TFs supported by at least three lines of evidence were included. To refine the list of potential TFs, a final manual curation step was performed to exclude false positives, mainly transcription-related proteins that were not actual TFs. These included members of the *Snf2* transcriptional regulator family (e.g., *Solyc01g067390*), type *IA topoisomerases* (e.g., *SlTOP3α, Solyc05g014720*), RNA polymerase II transcriptional co-activators (e.g., *Solyc08g082580*), and subunits of the chromatin remodeler *SWI/SNF* (e.g., *SlSWI3D, Solyc01g109510*). This resulted in a set of 1840 TFs (Supplemental Table 4). DNA-binding preferences were determined by retrieving position weight matrices (PWMs) from CisBP v.2 (Weirauch et al., 2014) or assigning PWMs from *Arabidopsis* and maize orthologs in JASPAR (Castro-Mondragon et al., 2022), yielding 846 TFs with assigned PWMs (Supplemental Table 4). This updated annotation and TF list served as the foundation for GRN construction in subsequent steps.

### Tomato genes exhibit widespread expression, but their expression levels vary across different organs

As a first step in organ-level GRN construction, we compiled a comprehensive dataset of publicly available tomato RNA-seq libraries (Supplemental Table 5). The transcriptomic libraries were categorized into five main organs: roots (1840 libraries from 124 studies), leaves (3778 libraries from 279 studies), flowers (568 libraries from 55 studies), fruits (4149 libraries from 147 studies), and seeds (270 libraries from 13 studies), for a total of 10 605 libraries that surpassed quality filters (Supplemental Figure 1A). These libraries were derived from a range of experimental contexts, including abiotic stress (3379 libraries), biotic interactions (2085 libraries), developmental studies (2872 libraries), and genetic modification studies (2035 libraries) (Supplemental Figure 1B). They also included a broad representation of tomato cultivars, with more than 120 genotypes. The most prevalent were M82 (16.7%), MicroTom (9.5%), and Ailsa Craig (7.3%), among others (Supplemental Figure 1C). This comprehensive dataset provided a robust foundation for construction of reference GRNs to address multiple research questions.

**Figure 1:**
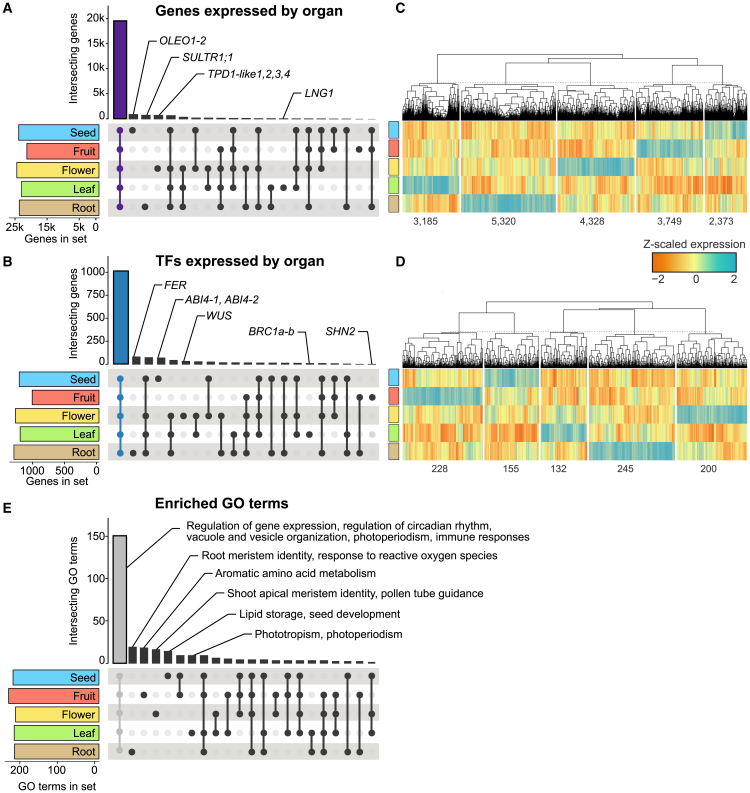
Organ-level transcriptomic landscape of tomato **(A)** Distribution of expressed genes across organs. **(B)** Distribution of expressed transcription factors (TFs) across organs. **(C)** Heatmap of normalized (*Z*-scaled) expression levels of shared genes across organs, indicating the number of genes in each cluster. **(D)** Heatmap of normalized (*Z*-scaled) expression levels of shared TFs across organs, indicating the number of TFs in each cluster. **(E)** Enriched biological process Gene Ontology (GO) terms associated with expressed genes across organs (adjusted *p* value <0.05).

Reads were mapped to gene models using the ITAG4.2-merged annotation, and genes with expression levels above 5 transcripts per million (TPM) in more than 10% of all libraries for a given organ were considered to be expressed in that organ. We identified 26 910 genes (71.82%) expressed in at least one organ, 19 361 (71.95%) of which were expressed in all organs. Thus, a minor fraction (15.9%) of expressed genes exhibited organ-level expression (Figure 1A and Supplemental Table 6). Similar expression patterns have been reported in maize, flaxseed, and wheat (Huang et al., 2018; Ramírez-González et al., 2018; Qi et al., 2023). Examples of organ-level genes include *Solyc06g051770* and *Solyc10g047720* in seeds, whose *Arabidopsis* homologs, *Oleosin 1* and *2* (*AtOLEO1-2*), are involved in seed oil body formation (Siloto et al., 2006). In roots, we identified *SlSULTR1*;*1* (*Solyc10g047170*), which encodes a sulfate transporter associated with external sulfate uptake (Takahashi et al., 2000). In flowers, *Tapetum Determinant 1-like* (*SlTPD1-like*) genes such as *Solyc11g005500*, *Solyc12g009850*, *Solyc05g010190*, and *Solyc04g071640* were specifically expressed, consistent with their role in tapetal cell development and gametogenesis (Ezura et al., 2017). In leaves, we found *Longifolia 1* (*SlLNG1*, *Solyc02g089030*), whose *Arabidopsis* homolog *AtLNG1* (*AT5G15580*) regulates leaf morphology by promoting longitudinal cell expansion (Lee et al., 2006) (Figure 1A).

Most TFs (1612, 87.7%) were expressed in at least one organ; 1014 (62.9%) were expressed across all organs, and a smaller subset (16.46% of expressed TFs) exhibited organ-specific expression (Figure 1B and Supplemental Table 6). This latter group included *SlBRC1a* and *SlBRC1b* (*Solyc03g119770* and *Solyc06g069240*), homologs of *Arabidopsis AtBRANCHED1* involved in leaf and axillary bud development (Martín-Trillo et al., 2011); *SlFER* (*Solyc06g051550*), a key regulator of root iron uptake (Aviña-Padilla et al., 2023); *SlWUSCHEL* (*SlWUS*, *Solyc02g083950*), which controls floral meristem identity and development (Hawar et al., 2022); *SlSHINE2* (*SlSHN2*, *Solyc12g009490*), whose encoded TF controls epidermal growth in developing fruits (Bres et al., 2022); and two paralogs of *Arabidopsis AtABI4* (*Solyc03g095977* and *Solyc03g095973*) that were exclusively expressed in seeds, consistent with their role in seed vigor (Bizouerne et al., 2021). Importantly, although most TFs and other genes were expressed across all organs, their expression levels varied substantially depending on the organ analyzed (Figure 1C and 1D). These quantitative differences suggest organ-level regulatory mechanisms, where distinct expression patterns contribute to the specialized functions and characteristics of each organ.

To assess how gene expression in tomato organs relates to the occurrence of organ-level and shared biological processes, we performed a gene set enrichment analysis (GSEA) for each organ. The analysis revealed that most enriched biological processes (151 GO terms) were common to all organs (adjusted *p* value <0.05); these included gene-expression regulation, circadian rhythm, vacuole and vesicle organization, immune responses, mRNA methylation, and response to abscisic acid (ABA) (Figure 1E and Supplemental Table 7). By contrast, only 22 enriched GO terms were identified as unique to specific organs. These included processes related to fruit ripening in fruits, root meristem identity and response to reactive oxygen species in roots, phototropism and photoperiodism in leaves, shoot apical meristem identity, brassinosteroid signaling, and pollen tube guidance in flowers, and lipid storage and seed development in seeds (Figure 1E, Supplemental Figure 2, and Supplemental Table 7). These findings demonstrate that although fundamental biological processes are conserved across all tomato organs, a subset of processes support unique organ-level functions, emphasizing the existence of a complex regulatory mechanism that defines organ identity in tomato.

### Organ-level GENIE3 networks recapitulate experimentally obtained TF–target interactions

**Figure 2:**
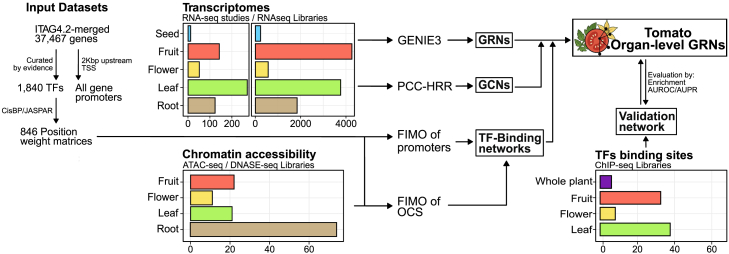
Multi-omics data integration for generation of organ-level GRNs in tomato Overview of datasets and processing steps used to generate organ-level gene-regulatory networks (GRNs) in *Solanum lycopersicum*. Bar plots indicate the number of available datasets per organ for transcriptomics (RNA-seq), chromatin accessibility (ATAC-seq/DNase-seq), and TF binding sites (ChIP-seq). Arrows illustrate data flow into regulatory network generation, including GRNs, co-expression networks (GCNs), TF binding networks, and validation datasets. TSS, transcription start site; PCC–HRR, Pearson correlation coefficient—highest reciprocal rankings; OCS, open chromatin site; AUROC, area under the receiver-operating characteristic curve; AUPR, area under the precision-recall curve; FIMO, Find Individual Motif Occurrences.

We compiled a comprehensive dataset of tomato omics data for GRN generation, including over 10 000 transcriptomes, nearly 100 chromatin accessibility experiments, and 16 ChIP-seq libraries (Figure 2). To generate organ-level GRNs, we used the GENIE3 algorithm with separate transcriptomic count tables for each organ and the updated TF list. GENIE3 generated a ranked list of putative TF–target interactions, from which we selected the top 1%, 2%, 5%, 8%, and 10% of the highest-scoring interactions to evaluate network accuracy. The inferred organ-level networks were benchmarked against ChIP-seq networks obtained from datasets for tomato TFs with more than 1000 mapped gene targets (Supplemental Table 8). These included data for *Sl*GLK1 and *Sl*GLK2 (Solyc07g053630, Solyc10g008160) (Tu et al., 2022), *Sl*MYC2 (Solyc08g076930) (Du et al., 2017), *Sl*JMJ4 (Solyc08g076390) (Ding et al., 2022), *Sl*WOX13 (Solyc02g082670) (Jiang et al., 2023), *Sl*EIL4 (Solyc06g073730), *Sl*TAGL1 (Solyc07g055920), and *Sl*RIN (Solyc05g012020) (Fujisawa et al., 2011; Gao et al., 2019) (Supplemental Table 8). Considering the ChIP-seq-derived networks as the gold standard, we assessed the quality of our GRNs by enrichment analysis using Fisher’s exact test. The analysis included the TFs *Sl*GLK1 and *Sl*GLK2, *Sl*MYC2, *Sl*EIL4, *Sl*JMJ4, and *Sl*WOX13 to evaluate all organs, but *Sl*RIN and TAGL1 were assessed exclusively in reproductive organs (excluding root and leaf networks), as their expression is restricted to these tissues. The top 2% networks showed the highest enrichment (odds ratio) and statistical significance (*p* values) in overlap between the TF–target pairs obtained by GENIE3 and those obtained by ChIP-seq (Table 1 and Supplemental Table 9). Furthermore, comparison with existing tomato gene networks from PlantRegMap (Tian et al., 2020) and TomatoNet (Kim et al., 2017) revealed that the GENIE3-derived GRNs exhibited greater enrichment and overlap with the gold-standard dataset, indicating better performance in prediction of experimentally obtained TF–target interactions (Supplemental Table 10).

**Table 1:** Enrichment metrics for organ-level GRNs

	Root	Leaf	Flower	Fruit	Seed
Total TFs	1297	1216	1300	1058	1241
Total genes	23 226	22 513	23 988	20 888	23 124
Total edges	797 120	743 902	798 851	665 733	817 504
Log_2_ Fisher odds ratio	1.89	2.66	2.01	3.01	2.22
Log_10_ adjusted *p* value	−156.72	−inf	−196.33	−305.17	−inf
Genes in overlap	1714	2967	1912	2226	4204
AUROC	0.72	0.65	0.65	0.72	0.53
AUPR	0.51	0.45	0.46	0.52	0.32

Summary of enrichment metrics for organ-level GRNs, considering the top 2% of interactions identified by the GENIE3 algorithm. Enrichment from a Fisher’s exact test (log_2_ odds ratio, −log_10_ adjusted *p* value, and intersection size) to the validation network (ChIP-seq). −inf represents log_10_ adjusted *p* values less than −400.

To further evaluate the performance of the top 2% GRNs, we calculated the area under the receiver-operating characteristic (AUROC) and area under the precision-recall (AUPR) curves for each organ-level network and compared these values to a network consisting of TF–target interactions obtained from the gold-standard dataset. The GRNs derived from tomato roots, leaves, flowers, fruits, and seeds had significantly higher AUROC and AUPR values than randomly generated TF–target pairs (Supplemental Figure 3). These results confirm that the GRNs successfully recapitulate experimentally validated TF–target interactions, underscoring their utility in predicting regulatory interactions for TFs that lack experimental validation.

To provide further support for our networks, we integrated additional layers of information from complementary approaches into the GENIE3-inferred edges. Gene co-expression is widely used to infer biologically relevant relationships between genes (Wolfe et al., 2005; Yin et al., 2021). Using the same RNA-seq datasets, we generated aggregated gene co-expression networks (GCNs) following the protocol in Orduña et al. (2023). The TF–target pairs from each GCN were extracted as additional evidence for the GENIE3-predicted interactions. Although the GENIE3 algorithm predicts regulatory interactions based on expression patterns, additional evidence is necessary to determine whether these interactions occur via direct TF binding to regulatory sequences. To integrate TF binding information into the GENIE3 networks, we extracted upstream sequences (2 kb upstream from the transcription start site [TSS]) for each annotated gene in ITAG4.2-merged and predicted TF binding sites using the Find Individual Motif Occurrences (FIMO) tool (Grant et al., 2011). In addition, we performed the same analysis on sequences within open chromatin sites (OCSs) identified in tomato fruits, flowers, leaves, and roots using data from DNase-seq and ATAC-seq experiments (Supplemental Table 11). We found that most of the OCSs were organ specific, indicating a high degree of specialization in chromatin accessibility among tomato organs (Supplemental Figure 4). Fruits exhibited the largest number of unique OCSs, suggesting extensive regulatory activity, whereas shoots had the lowest overall OCS abundance. Notably, flowers and fruits shares a substantial proportion of accessible regions, reflecting a high similarity in euchromatic regions in tomato reproductive organs (Supplemental Figure 4).

**Figure 3:**
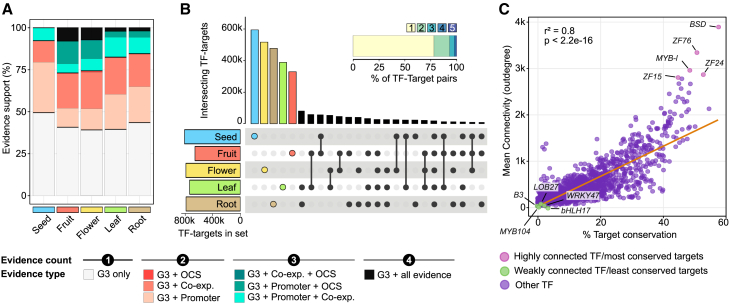
Comparative analysis of organ-level GRNs reveals regulatory signatures and TF connectivity patterns **(A)** Stacked bar plot showing the proportion of TF–target pairs in the GENIE3-inferred GRNs supported by multiple sources of evidence. Bar colors show interactions validated by GENIE3 alone (G3) or by 2–4 sources, including open chromatin site (OCS) binding, gene co-expression networks (Co-exp.), and promoter binding. **(B)** UpSet plot displaying the overlap of TF–target interactions across organ-level GRNs, with an inset showing the distribution of shared versus unique interactions (1–5 organs). **(C)** Relationship between TF mean connectivity (average number of target genes across organs) and target conservation (percentage of shared targets across organs). Dot colors indicate TF groups by connectivity/conservation of targets. An orange trend line highlights the general pattern in the data.

The results from all evidence layers were compiled, ensuring that our analysis remained constrained to TF–target pairs identified by GENIE3. This approach maintained the predefined network structure and mapped additional regulatory evidence onto it, rather than introducing new interactions. Between 51% and 61% of the GENIE3-predicted interactions were supported by at least one additional piece of evidence, and most edges were confirmed by one or two different approaches (Figure 3A). Furthermore, a substantial portion of GENIE3 edges were confirmed by the presence of *cis*-binding motifs detected by FIMO within promoter sequences (from 28% to 30% of the edges), suggesting potential direct regulation of TFs over their inferred targets.

As mentioned previously, most genes and TFs were expressed across all tomato organs, although at varying levels. To assess how these expression patterns influence regulatory interactions, we analyzed the distribution of TF–target gene pairs across the five organ-level GRNs. Notably, over 75% of these pairs were unique to a single organ (Figure 3B), indicating that although TFs and targets are broadly expressed, regulatory interactions are largely organ specific. This result was expected, as it is consistent with findings from other plant GRNs (Huang et al., 2018; Ranjan et al., 2024), supporting the biological validity of our networks. As discussed above, the majority of OCSs (which define the potential for TF binding) are organ dependent (Supplemental Figure 4), indicating that chromatin accessibility is a relevant determinant of organ-level gene regulation. Nonetheless, additional layers of regulation may also influence the presence or absence of TF–target interactions, including post-transcriptional control of TF protein levels, post-translational modifications required for TF activity or subcellular localization (e.g., phosphorylation triggering nuclear localization or retention), and the availability of co-factors or partner TFs.

To further evaluate how specific TF–target pairs are distributed across organs and how target conservation correlates with TF connectivity (node outdegree), we calculated the percentage of conserved targets for each TF across all organs. As expected, most TFs showed a low percentage of conserved targets owing to the organ-level nature of TF–target interactions. Interestingly, we observed a significant correlation between TF connectivity and target conservation (*R*^2^ = 0.36, *p* < 2.2e−16), indicating that highly connected TFs tend to regulate the same target genes across organs (Figure 3C). GSEA of the top five most connected TFs with conserved targets (MYB-l Solyc01g079210, ZF15 Solyc01g110490, ZF24 Solyc03g026350, BSD Solyc10g005900, and ZF76 Solyc12g017410) revealed strong enrichment of GO terms related to fundamental processes, including nucleic acid metabolism, vesicle transport, and RNA metabolism (false discovery rate [FDR]-adjusted *p* value <0.05) (Supplemental Figure 5). These results suggest that TFs with high connectivity and conserved targets function as global regulators of essential pathways. By contrast, TFs with lower connectivity and limited target conservation appear to mediate more specific functions. For instance, despite their ubiquitous expression, TFs such as *LOB27* (*Solyc06g062630*) and *bHLH17* (*Solyc02g093280*) show enriched functions in flowers, *WRKY47* (*Solyc01g058540*) in roots, and *B3* (*Solyc05g004000*) in flowers and seeds, whereas *MYB104* (*Solyc01g090530*) exhibits distinct functions across multiple organs (Supplemental Figure 6). In summary, organ-level GENIE3 networks present topological features expected for GRNs and can recapitulate TF–target interactions obtained experimentally by complementary approaches.

### The fruit GRN captures known regulatory interactions and identifies novel central controllers of ripening

**Figure 4:**
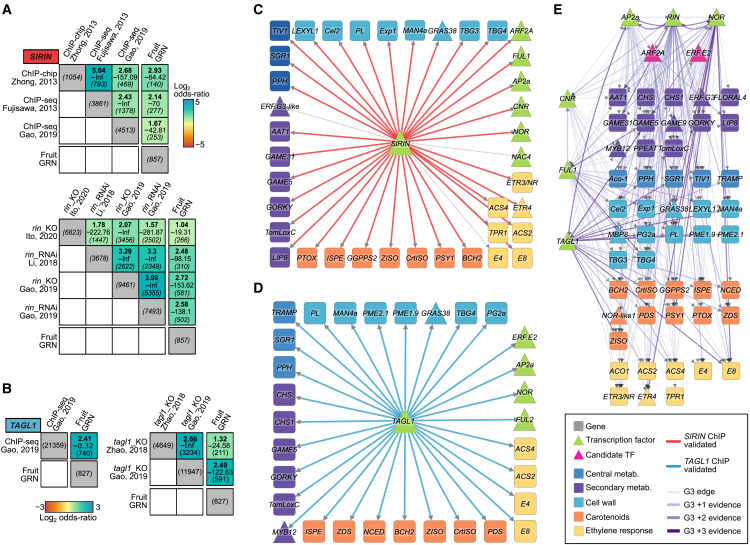
Identification of key transcriptional regulators of fruit-ripening regulatory cascades in tomato **(Aand B)** Enrichment and validation of fruit GRNs for *SlRIN***(A)** and *TAGL1***(B)** using knockout-mutant data and ChIP-binding analyses. Box heatmaps display enrichment results from a Fisher’s exact test (log_2_ odds ratio, −log_10_ adjusted *p* value, and intersection size). **(Cand D)** Network representation of ripening-associated genes (Li et al., 2019; Zhu et al., 2022a) for *SlRIN***(C)** and *TAGL1***(D)** derived from the fruit GRN. Triangles represent TFs, and squares represent target genes. Node colors indicate function. Edges are colored red (*SlRIN*) or blue (*TAGL1*) when interactions are validated by ChIP-binding evidence. **(E)** Network of key regulators of ripening-associated genes (top-scored TFs), using the same node and edge color scheme as in **(C) and (D)**. Pink nodes are candidate key TFs. Darker edge shades indicate accumulated regulatory evidence.

To evaluate the ability of the organ-level GRNs to capture biologically relevant regulatory interactions, we focused on ripening, one of the most extensively studied processes in tomato linked to hormonal signaling pathways such as ethylene, cell wall remodeling, pigment biosynthesis, and other processes (Karlova et al., 2014; Li et al., 2019; Zhu et al., 2022a). Tomato fruit ripening is governed by a complex regulatory cascade involving epistatic interactions between well-characterized TFs, including APETALA2a (*Sl*AP2a), NON-RIPENING (*Sl*NOR), FRUITFULL (*Sl*FUL1/TDR4 and *Sl*FUL2/MBP7), TOMATO AGAMOUS-LIKE 1 (TAGL1), RIPENING INHIBITOR (*Sl*RIN), and COLORLESS NON-RIPENING (*Sl*CNR) (Li et al., 2021a; Zhu et al., 2022a). Among these, TAGL1 and *Sl*RIN are recognized as central regulators of ripening (Gao et al., 2019; Li et al., 2021a; Li et al., 2021b). To determine whether the fruit GRN reproduced regulatory interactions of known TFs, we compared the targets of TAGL1 and *Sl*RIN (TFs predominantly expressed in fruits) from the fruit GRN with gene lists compiled from previous omics studies. These included differentially expressed genes (DEGs) identified in *TAGL1* and *SlRIN* knockout and RNAi plants (Li et al., 2018; Gao et al., 2019; Ito et al., 2020), as well as direct binding targets identified via *Sl*RIN ChIP-chip and ChIP-seq (Fujisawa et al., 2013; Zhong et al., 2013; Gao et al., 2019) and TAGL1 ChIP-seq (Gao et al., 2019) experiments. For *Sl*RIN, we observed a statistically significant overlap between the targets identified in the fruit GRN and targets obtained in all the experiments, including ChIP-binding targets and regulatory targets identified in *Sl*RIN-deficient plants (Figure 4A). Similarly, for TAGL1, the fruit GRN identified targets validated by ChIP-seq and/or knockout experiments (Figure 4B). Many of these correspond to direct TF binding to target promoters, with 45% of *Sl*RIN GRN targets (411/857) and 89% of TAGL1 GRN targets (740/827) validated by ChIP-binding evidence (Figure 4A and 4B). These findings highlight the potential of the GENIE3 GRN to capture experimentally validated regulatory interactions.

To further evaluate the regulatory roles of *Sl*RIN and TAGL1 in fruit ripening, we generated *Sl*RIN and *Sl*TAGL1 subnetworks, focusing on genes involved in fruit ripening described in Li et al. (2019) and Zhu et al. (2022a). The GENIE3 network predicted interactions of both TFs with a large number of ripening genes (Supplemental Table 13). Furthermore, most of the GENIE3 edges that connect *Sl*RIN and TAGL1 to these targets (90% for *Sl*RIN and 100% for TAGL1) are supported by ChIP evidence (Figure 4C and 4D). In addition, the subnetworks highlight the regulatory influence of both TFs across diverse biological processes, including ethylene signaling, ABA signaling, and cell wall modification, as well as their interactions with other known TFs in fruit ripening, including *Sl*CNR, *Sl*NOR, and *Sl*AP2a (Figure 4C and 4D).

To identify novel ripening regulators, we used the fruit GENIE3 network to generate a subnetwork that included the list of ripening-associated genes (Li et al., 2019; Zhu et al., 2022a). To quantify network hub influence, we used the integrated value of influence (IVI), a metric that combines measures of degree centrality, cluster rank, neighborhood connectivity, betweenness centrality, and collective influence into a single value (Salavaty et al., 2020). We found that the TFs *Sl*CNR, *Sl*NOR, *Sl*FUL1, *Sl*AP2a, *Sl*RIN, and TAGL1 were among those with the highest IVI, confirming their roles as central regulators of fruit-ripening genes. Two additional TFs, *Sl*ARF2A (Solyc03g118290) and *Sl*ERF.E2 (Solyc06g063070), emerged with high IVIs, suggesting that they may play roles in controlling ripening-related genes (Supplemental Table 14). The *Sl*ARF2A TF has been identified as a regulator of axillary shoot development and is predominantly expressed in the late stages of ripening (Xu et al., 2016). RNAi lines targeting *Sl*ARF2A exhibit ripening defects and ethylene insensitivity, whereas overexpression lines show accelerated and uneven ripening (Hao et al., 2015; Breitel et al., 2016). We found that the predicted targets of *Sl*ARF2A in our GRN were significantly enriched among the regulatory targets of *Sl*ARF2A identified using overexpressor plants (Breitel et al., 2016) and *SlARF2A* RNAi lines (Hao et al., 2015). Notably, predicted targets of *Sl*ARF2A also included a high proportion of ripening-related genes (Supplemental Figure 7A). Among them, we recovered important genes involved in ethylene signaling, including *SlACS4* (*Solyc05g050010*), *SlE8* (*Solyc09g089580*), and *SlETR3-4* (*Solyc09g075440*), and carotenoid metabolism, including *SlPSY1* (*Solyc03g031860*), *SlPDS* (*Solyc03g123760*), and *SlZDS* (*Solyc01g097810*). In addition, *SlNOR* (*Solyc10g00680*), *SlAP2a* (*Solyc03g044300*), *SlCNR* (*Solyc02g077920*), and *SlFUL1* (*Solyc06g069430*) were also identified as targets, consistent with previous reports (Hao et al., 2015; Breitel et al., 2016) (Supplemental Figure 7B). These results confirm the biological relevance of *Sl*ARF2A predicted by our GRN and reinforce its potential key role in the regulation of fruit ripening. Conversely, the role of *Sl*ERF.E2 in ripening has not yet been characterized. Interestingly, we found that *Sl*ARF2A and *Sl*ERF.E2 may act upstream of important TFs such as *Sl*AP2, *Sl*NOR, and *Sl*CNR, in addition to multiple ripening-relevant genes (Figure 4E).

### Tomato organ-level GRNs confirm the role of ABF TFs in ABA regulatory cascades and identify *Sl*GBF3 as a new regulator of ABA-related genes

**Figure 5:**
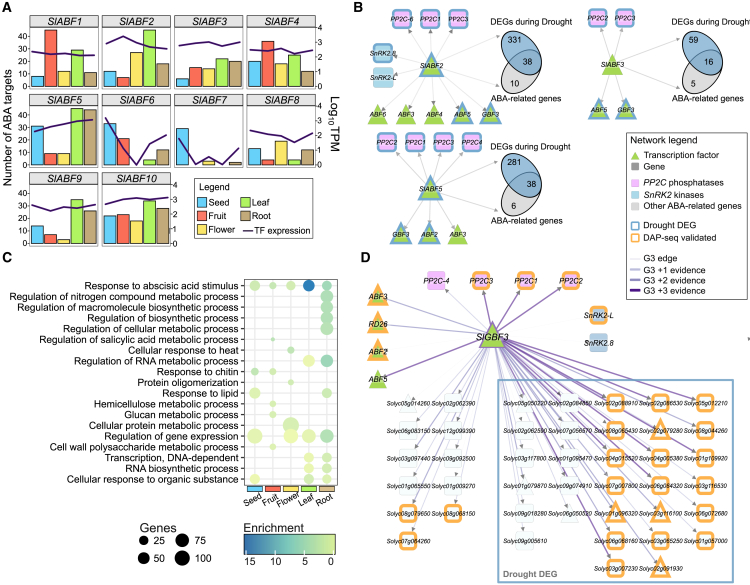
Tomato GRNs reveal the role of ABF TFs and identify a key regulator of ABA-related GRNs **(A)** The numbers of ABA-related targets for *SlABF* TFs are shown as bars. The mean expression of each TF is superimposed as a black line in log_10_ TPM. **(B)** Enrichment ratio and validation of the leaf GRNs for *SlABF3* and *SlABF5* using drought-responsive DEGs (Wang et al., 2023b). Box heatmaps (left) display enrichment results from a Fisher’s exact test (log_2_ odds ratio, −log_10_ adjusted *p* value, and intersection size). The networks (right) show the distribution of ABA-related and drought-regulated targets of these TFs. **(C)** Gene set enrichment analysis (GSEA, FDR-adjusted *p* value < 0.05) of the *SlGBF3* target genes in organ-level GRNs. Dot size represents gene number, and color intensity reflects enrichment value. **(D)** Network visualization of *SlGBF3*-regulated ABA-related genes in the leaf GRN. Triangles represent TFs, and squares represent target genes. Node colors indicate function. Orange-bordered nodes indicate DAP-seq validated genes, and enclosed nodes are DEGs from drought-stressed leaves (Wang et al., 2023b). Darker edge shades indicate accumulated regulatory evidence.

Notably, the GO term “response to ABA” was consistently enriched across all organs (Supplemental Figure 2). This category includes 730 genes, 714 of which are ubiquitously expressed in tomato organs (Supplemental Table 15, hereafter referred to as “ABA-related genes”). Known TFs that participate in ABA signaling include members of the AREB/ABF (ABA response element binding/ABA response element binding factor) family of bZIP TFs (Uno et al., 2000; Krukowski et al., 2023). This family has ten members in tomato (Pan et al., 2023); however, the relative contribution of each ABF TF at the organ level remains unexplored. To address this, we constructed organ-level networks for the ten *Sl*ABFs, focusing specifically on their regulation of ABA-related genes. Although most *Sl*ABF family members are expressed at similar levels across organs—with the exception of *SlABF6* (not expressed in flowers) and *SlABF7* (not expressed in fruits and leaves)—their regulatory potential differs depending on the organ. *Sl*ABF1 and *Sl*ABF4 appear to play key roles in the regulation of ABA-related genes in fruits, whereas *Sl*ABF2, *Sl*ABF3, *Sl*ABF5, and *Sl*ABF10 regulate more genes in the leaf network. *Sl*ABF5, *Sl*ABF9, and *Sl*ABF10 are involved in ABA-response regulation in roots, whereas *Sl*ABF2 appears to play a more important role in the leaf and flower GRNs. In seeds, *Sl*ABF6 and *Sl*ABF7 exhibit the highest regulatory activity on ABA-related genes (Figure 5A and Supplemental Table 16). These findings indicate that the ABF TFs make different relative contributions to the regulation of ABA-related genes across organs.

Plant responses to drought stress are tightly regulated by ABA signaling pathways (Kang et al., 2002; Krukowski et al., 2023). To assess the regulatory relevance of *Sl*ABF TFs in the drought response, we focused on the leaf-specific GRN and extracted all the predicted targets of each *Sl*ABF. These target sets were compared with a list of DEGs from water-stressed tomato leaves (Wang et al., 2023b) to identify significant overlaps between lists. We found three *Sl*ABFs that had significant overlaps (thresholds: two-fold enrichment and *p* < 0.01) with the drought-responsive genes: *Sl*ABF2 (369 of 607 targets, 61%), *Sl*ABF3 (75 of 168 targets, 45%), and *Sl*ABF5 (319 of 410 targets, 78%) (Supplemental Figure 8 and Supplemental Table 17). Although other *Sl*ABFs (with the exception of *Sl*ABF4) met the statistical cutoff, their enrichment levels were low, suggesting a more limited or secondary role in the drought response of leaves. Consistent with our results, *Sl*ABF3 and *Sl*ABF5 have been previously implicated in drought responses of tomato (Hsieh et al., 2010; Orellana et al., 2010), and *Sl*ABF2 is known to be drought inducible (Fuentes-Merlos et al., 2023; Wexler et al., 2024). Further analysis revealed that a substantial proportion of ABA-related targets of these TFs were also drought responsive: 80% for *Sl*ABF2 (39/49), 73% for *Sl*ABF3 (16/22), and 86% for *Sl*ABF5 (38/45) (Figure 5B and Supplemental Table 17). Network visualization suggested that these TFs may regulate key components of the ABA signaling pathway, particularly members of the *protein phosphatase 2C* (*PP2C*) gene family. For instance, *PP2C40* (*Solyc03g121880*) is regulated by all three TFs, *PP2C28* (*Solyc03g096670*) by *Sl*ABF2 and *Sl*ABF5, *PP2C30* (*Solyc05g052980*) by *Sl*ABF3 and *Sl*ABF5, and *PP2C60* (*Solyc07g062970*) and *PP2C52* (*Solyc06g076400*) by *Sl*ABF2 and *Sl*ABF5, respectively. In addition, *Sl*ABF2 targets two *SnRK2* kinases, *SnRK2*.*8* (*Solyc04g012160*) and *SnRK2-L* (*Solyc08g077780*) (Figure 5B). Interestingly, we observed potential regulatory interactions among these TFs, including reciprocal regulation between *Sl*ABF3 and *Sl*ABF5, as well as evidence that *Sl*ABF2 may regulate both *Sl*ABF3 and *Sl*ABF5, suggesting a hierarchical structure (Figure 5B). Together, these results support a central role for *Sl*ABF2, *Sl*ABF3, and *Sl*ABF5 in orchestrating the ABA-mediated drought response in tomato.

To identify novel regulators of ABA responses beyond the *Sl*ABF family of TFs across all organs, we filtered the five organ-level GRNs to retain TFs with regulatory connections to ABA-related genes. A network analysis calculating the IVI of the network hubs identified the *Sl*GBF3 TF (Solyc01g095460) as one of the top ten most influential TFs in the ABA-related networks across all organs (Supplemental Table 18), consistent with recent evidence showing that it is co-expressed with drought-responsive genes in tomato leaves (Bortolami et al., 2024). Several other TFs were highly ranked regulators shared across organ-level GRNs. *Sl*WRKY31 (Solyc06g066370) ranked first in the fruit and seed networks, fourth in flowers, and tenth in leaves and roots. *Sl*WRKY31 physically interacts with the *Sl*VQ15 TF to cooperatively regulate defense against *Botrytis cinerea* and plays a role in fruit ripening downstream of *Sl*WD40 (Zhu et al., 2022b; Huang et al., 2022). Although a direct role in ABA responses has not been established, *Sl*WRKY31 is upregulated in response to different stresses, including drought, salt, and pathogen infection (Huang et al., 2012), suggesting that it may contribute to stress-responsive transcriptional programs. *Sl*NAC/JA2L (Solyc07g063410) was ranked second in roots, fourth in leaves, and seventh in flowers. JA2L acts downstream of MYC2 in the jasmonate-mediated response to *B. cinerea* and regulates jasmonate-mediated stomatal reopening during *Pseudomonas syringae* infection (Du et al., 2017). JA2L is slightly induced by ABA and strongly induced by dehydration (Huang et al., 2012; Dong et al., 2023), suggesting a potential role in crosstalk between the ABA and jasmonate pathways. We also identified the zinc finger transcription factor 47 (Solyc06g072720), although its function remains uncharacterized. Most of the remaining top TFs were either shared between two organs or specific to a single organ. These include known components of the ABA signaling pathway such as *Sl*ABF5, ranked first in roots and leaves, and *Sl*ABF2, ranked fifth in leaves, consistent with their potential central role in ABA and drought GRNs (Figure 5B). Additional ABA-related TFs were found among the top regulators in seeds, including *Sl*ABI3-2 (Solyc06g083590) and *Sl*ABI4-2 (Solyc03g095977), orthologs of the key seed development regulators *At*ABI3 and *At*ABI4 (Bizouerne et al., 2021). We also identified Solyc05g050220, a second member of the G-box binding factor (GBF) family and a close homolog of *At*GBF3, similar to *Sl*GBF3 (Bortolami et al., 2024). This TF is upregulated under drought stress (Dong et al., 2023) and was ranked third in roots and eighth in leaves, suggesting possible functional redundancy with *Sl*GBF3 in these organs.

To further explore the role of *Sl*GBF3, we performed a GSEA on its target genes in each organ-level GRN. We found significant enrichment of genes associated with the “response to abscisic acid stimulus” biological process shared across all organ networks, indicating a potential conserved role in the regulation of ABA-related genes (Figure 5C). To further validate the potential regulatory interactions of *Sl*GBF3 identified in the GRNs, we performed DAP-seq. The DAP-seq analysis identified ∼13 000 consensus binding events (peaks) in genomic regions of both experimental replicates and enriched over the negative control. These peaks were associated with 11 561 genes from the ITAG4.2-merged annotation, including multiple ABA-related genes such as *SlHOX6* (*Solyc01g096320*), *SlABF3* (*Solyc01g108080*), *SlPP2C1* (*Solyc03g121880*), and *SlRAF*, which encodes a raffinose synthase (*Solyc02g086530*) (Supplemental Table 19 and Supplemental Figure 9A). Using the identified peak regions, we determined the DNA-binding motif of *Sl*GBF3 through a MEME-chip analysis (Machanick and Bailey, 2011). Our analysis revealed that *Sl*GBF3 predominantly binds to the consensus sequence “AYGTGGCA” (Supplemental Figure 9B), which is similar to the motifs described for *At*GBF3 in the Cis-BP (Weirauch et al., 2014) and JASPAR (Castro-Mondragon et al., 2022) databases (MA1351.1-3, with an average consensus sequence of “CGTGGCA”) (Supplemental Figure 9B). Accordingly, the binding motif of *Sl*GBF3 was significantly correlated (Pearson correlation coefficient 0.7 – 0.865) with the motifs of *At*GBF3 (Supplemental Figure 9C), indicating strong evolutionary conservation between these TFs and validating our experimental results.

To evaluate the functional relevance of the identified binding events, we compared the predicted targets in the tomato organ-level GRNs with those identified through DAP-seq. Genes that overlapped between these datasets were classified as high-confidence targets (HCTs). Our analysis revealed that 42%–58% of the GRN-predicted targets of *Sl*GBF3 were supported by DAP-seq binding evidence, with this overlap being significantly enriched (Supplemental Figure 10).

To further explore the role of *Sl*GBF3 in ABA-related regulatory networks, we generated GRNs specifically for ABA-responsive genes across different organs. The leaf-specific GRN exhibited the highest representation of ABA-related genes. Notably, over 60% of the *SlGBF3* targets in the leaf network were associated with drought stress responses, including multiple *PP2C* genes (*PP2C28*, *PP2C30*, *PP2C40*, and *PP2C60*) and two *SNF1-related protein kinase 2* (*SnRK2*) genes (*SnRK2-L* and *SnRK2*.*8*) (Figure 5D). In addition, *Sl*GBF3 appears to function as an upstream regulator of key TFs that control ABA-related genes, including *SlABF2*, *SlABF3*, and *SlABF5*. In the leaf network, over 50% of ABA-related targets were classified as HCTs on the basis of DAP-seq evidence (Figure 5D). A GSEA of the complete network of HCTs further revealed significant enrichment in regulatory functions related to water deprivation, abiotic stimuli, and hormone responses, with ABA as a central component (Supplemental Figure 11). Finally, to explore how *Sl*GBF3 binding could affect the expression of its target genes, we examined the expression profiles of *Sl*GBF3 and its HCTs in different available transcriptomic studies that evaluated gene-expression responses to drought in tomato leaves (Supplemental Table 20), focusing in particular on ABA-related target genes determined by our leaf GRN. We found that most of the target genes were upregulated during drought treatments across multiple datasets. Notably, *Sl*GBF3 was consistently induced under drought conditions, suggesting that it is a direct activator of ABA-related targets (Supplemental Figure 12). In GRNs of non-leaf organs, *Sl*GBF3 regulated smaller subsets of ABA-responsive genes. These included *SlPP2C* genes, *ABF* TFs, and key regulators such as *SlMYB1* (*Solyc12g099120*), a TF implicated in ABA-mediated pathogen susceptibility (Abuqamar et al., 2009) (Supplemental Figure 13). Although the leaf-specific GRN contained the largest number of ABA-related targets, all organ-level ABA networks contained a consistent proportion of HCTs (∼50%) (Supplemental Figure 13). This result suggests a conserved regulatory role of *Sl*GBF3 across different organs, reinforcing its significance in ABA-mediated stress responses.

### TomViz rGRN app: An online tool for visualization of tomato GRNs

**Figure 6:**
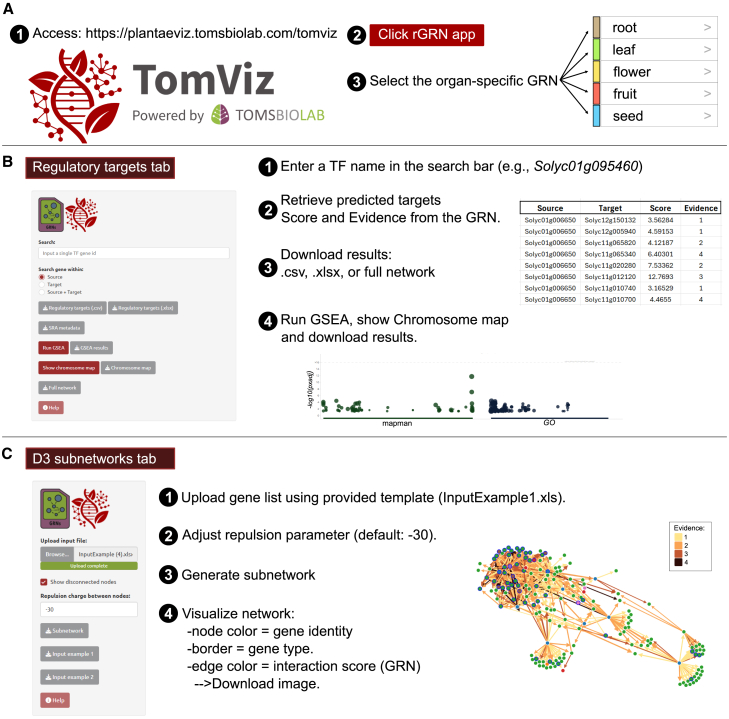
TomViz-GRNs: A web-based platform for exploring tomato organ-level GRNs **(A)** TomViz interface within the PlantaeViz platform (Santiago et al., 2024), providing access to GRN exploration tools. **(B)** Regulatory targets tab: users can query TFs or genes to explore regulatory interactions and validation layers. The interface includes options to download TF target lists, perform GSEA, and visualize target distributions on a chromosome map. **(C)** D3 subnetwork tab: users can upload gene lists, visualize GRNs, and analyze regulatory pathways at the organ level. The visualization includes directional edges representing regulatory interactions from TFs to targets, with edge colors indicating the level of supporting evidence. Additional options enable customization of network layout and node separation.

To provide the scientific community with a comprehensive framework of organ-level tomato GRNs and a user-friendly resource, we developed a public web platform featuring an interactive interface that enables users to explore the results of this study. Our reference GRN (rGRN) app is available within the TomViz module of the PlantaeViz platform (https://plantaeviz.tomsbiolab.com/tomviz) and adheres to the findability, accessibility, interoperability, and reusability (FAIR) principles (Santiago et al., 2024). Through the website, users can access the platform, launch the rGRN app, and select the organ-level network of interest (Figure 6A). Within the regulatory targets tab, users can enter a TF of interest to retrieve its predicted targets, along with interaction scores and evidence of interaction derived from the GRN. Additional tools enable users to perform a GSEA, visualize target chromosomal positions, and download result files in various formats (Figure 6B). In the D3 subnetworks tab, users can upload a gene list using a provided template, adjust network layout parameters such as node repulsion, and generate a subnetwork visualization based on interaction evidence on the GRN. Nodes are annotated by gene identity and type, and the resulting network can be exported for downstream analysis (Figure 6C). Integrating all our analyses, the TomViz rGRN app provides an intuitive platform for studying tomato gene regulation and investigating stress responses across different organs.

## Discussion

### Bioinformatic validation of GRNs

To construct tomato GRNs, we first generated an updated resource of gene models, TFs, and functional annotations. Existing tomato gene lists differ greatly across different studies, limiting exhaustive analyses of tomato regulatory cascades. To address this issue, we compiled and integrated gene annotations from the latest tomato genome assembly (SL4.0) into ITAG4.2-merged. Using well-established gene-annotation pipelines (Jones et al., 2014; Cantalapiedra et al., 2021), we assigned functional annotations to 24 356 of the 37 467 ITAG4.2-merged genes, considerably improving coverage compared with the previous ITAG4.1 annotation in SolGenomics, which included only 13 142 functionally annotated genes (Fernandez-Pozo et al., 2015), and similar to a previous annotation reported for iTAG4.0 (25 285 genes) (Rivera-Silva et al., 2024).

TF prediction remains a challenging task, as automated approaches usually rely on scanning protein sequences for known DNA-binding domains, which may lead to the inclusion of proteins with DNA-binding capabilities unrelated to TF function (Itzkovitz et al., 2006; Liebold et al., 2024). To avoid such proteins and refine the tomato TF list, we integrated multiple levels of evidence to filter and extract a curated set of 1840 TFs (representing around 5% of tomato genes). This number closely aligns with TF counts reported for tomato in PlantTFDB (1845 TFs) (Jin et al., 2017) and is slightly higher than those in CisBP (1773 TFs) (Weirauch et al., 2014) and other studies (1069 TFs in Kumar et al., 2021). Overall, the percentage of TF-encoding genes we found is slightly lower than that reported for *Arabidopsis* (approximately 5%–10%) (Riechmann and Ratcliffe, 2000) but consistent with estimates for other crops, such as wheat (5.7%) and rice (6.1%) (Zheng et al., 2016). It is also comparable to values reported for other Solanaceae species, such as eggplant (5.3%) (Wei et al., 2020). Our efforts provide a comprehensive framework to support the development of genomic tools for studying tomato regulatory cascades.

A vast number of transcriptomic studies have been conducted in tomato, covering diverse experimental conditions, organs, and developmental stages. Several efforts have aimed to generate gene-expression atlases for tomato (Ozaki et al., 2010; Fukushima et al., 2012; Gao et al., 2013; Koenig et al., 2013; Arhondakis et al., 2016; Zouine et al., 2017; Bae et al., 2021; Bizouerne et al., 2021; Kumar et al., 2021; Kusano et al., 2022; Li et al., 2024), but many studies have been limited in scope, often focusing on specific experimental conditions, using outdated genome assemblies (SL2.5 or SL3.0 with iTAG2.4 or iTAG3.0 annotations), or relying on microarray data. To our knowledge, the gene-expression dataset collected in this study represents the most comprehensive to date, compiling over 10 000 RNA-seq libraries from five major organs and integrating hundreds of bioprojects performed worldwide. Moreover, the transcriptomes were processed using the latest genome version (SL4.0) and the updated ITAG4.2-merged annotation, resulting in greater gene coverage. This extensive dataset enabled us to characterize general gene-expression patterns at the organ level, facilitating the identification of genes involved in organ-level functions. Organ identity has been shown to be the strongest determinant of differential gene expression, surpassing other experimental variables and highlighting the role of developmental processes in shaping transcriptome profiles (Aceituno et al., 2008). Thus, consistent with previous studies (Li et al., 2024), we found that most tomato genes met the threshold for expression across all organs. Similar ubiquitous expression patterns have been reported in other plants, including *Linum usitatissimum* (Qi et al., 2023) and *Zea mays* (Huang et al., 2018), in which over 50% of genes are expressed across multiple tissues.

In addition to broadly expressed genes, we also identified a substantial subset of genes with organ-level expression that were enriched in biological processes critical for organ function, including several genes previously shown to have organ-level expression (Siloto et al., 2006; Martín-Trillo et al., 2011; Ezura et al., 2017; Bizouerne et al., 2021; Bres et al., 2022; Hawar et al., 2022; Aviña-Padilla et al., 2023; Li et al., 2024). Furthermore, the TFs also displayed widespread expression across tomato organs, mirroring findings in *Arabidopsis* (Ranjan et al., 2024). Accordingly, prior studies in tomato have shown that fewer than 20% of expressed TFs are organ specific (Rohrmann et al., 2012). Nonetheless, despite their broad expression, TFs showed variation in expression levels across organs, highlighting the dynamic and context-dependent regulation of the transcriptional networks that govern organ function.

Transcriptomic data have been used extensively to generate biological network models with the purpose of identifying key candidates for functional genomics analyses. Because a limited amount of TF–target interaction data have been available for tomato, the majority of such studies have relied on GCNs to infer regulatory relationships and identify co-regulated gene groups (Fukushima et al., 2012; Koenig et al., 2013; Ichihashi et al., 2014; Arhondakis et al., 2016; Yue et al., 2016; Kim et al., 2017; Zouine et al., 2017; Bizouerne et al., 2021; Kusano et al., 2022; Manosalva and Vandepoele, 2023; Pirona et al., 2023; Wang et al., 2023a). GCNs lack directionality, making it difficult to establish regulatory interactions. In addition, many rely on correlations such as Pearson coefficients, which fail to capture non-linear relationships (Escorcia-Rodríguez et al., 2023). To address these limitations, we employed GENIE3, a widely used algorithm for reconstruction of directed GRNs in plants (Huang et al., 2018; Harrington et al., 2020; Tu et al., 2020; De Clercq et al., 2021; Chen et al., 2023; Ranjan et al., 2024) and other organisms (Huynh-Thu et al., 2010; Huynh-Thu and Geurts, 2019; Cuesta-Astroz et al., 2021; Olivares-Yañez et al., 2021). GENIE3 requires only gene-expression data as input, making it particularly suitable for tomato, in which TF gene targets remain poorly characterized. To validate our GRNs, we benchmarked them against available ChIP-seq data standard networks, using AUPR and AUROC curve values. This strategy, previously used to assess GRN performance in plants (Brooks et al., 2019; Contreras-López et al., 2022), provides a more centered evaluation of TF–target interactions than alternative methods that use gene co-association to biological processes or metabolic pathways (Kim et al., 2017; Orduña et al., 2023). Our analysis revealed that networks constructed from the top 2% TF–target scoring pairs yielded the best performance when evaluated against gold-standard datasets. These networks contained between 660 000 and 800 000 edges, consistent with previous GENIE3-based GRN studies in crops, in which networks typically included approximately 1 million edges (Huang et al., 2018; Ramírez-González et al., 2018; Harrington et al., 2020). The GENIE3-derived GRNs outperformed other tomato networks from public resources such as PlantRegMap (Tian et al., 2020) and other genome-scale biological network models (Kim et al., 2017). In addition, more than 50% of GENIE3-predicted edges were supported by one or more pieces of independent evidence, including *cis*-regulatory motif binding predictions, further reinforcing the biological relevance of these regulatory connections, as shown in other networks (De Clercq et al., 2021; Chen et al., 2023). This integration of multiple validation strategies enhances the accuracy and functional significance of inferred GRNs, providing a robust framework for studying transcriptional regulation in tomato.

Our analysis revealed that although many genes are broadly expressed across tomato organs, most of the TF–target interactions were organ specific. This contrasting result suggests that gene expression alone is insufficient to explain regulatory specificity and highlights the importance of additional layers acting in the cells. This phenomenon has been observed for GRNs in other species (Huang et al., 2018; Ranjan et al., 2024). A potential explanation may be organ-level chromatin accessibility, as we found that tomato showed a strong organ-level distribution of OCSs, suggesting that TF binding and regulatory activity are potentially constrained by the chromatin landscape of each organ. This has been reported previously at the organ level (Zhang et al., 2012; Tannenbaum et al., 2018) and even between different cell types of the same organ (Maher et al., 2018; Dorrity et al., 2021; Marand et al., 2021; Feng et al., 2022). Together, these results underscore the complexity of transcriptional regulation in plant systems and highlight the value of integrating chromatin accessibility, transcriptomic data, and network-based models to capture the context-dependent nature of gene regulation.

We identified a positive correlation between TF connectivity and target conservation across organs. Highly connected TFs (hubs) tended to regulate a similar set of targets in all organs, whereas TFs with fewer connections were more likely to control organ-level processes. For example, *SlHUA* (*Solyc12g017410*) primarily regulates genes involved in core cellular functions, whereas *SlNGA3* (*Solyc05g004000*) exhibits distinct, tissue-specific roles, including the regulation of auxin response in flowers and defense-related genes in seeds. Although *SlNGA3* is homologous to *AtNGA3*, a TF associated with flower development in *Arabidopsis* (Salava et al., 2022), our analysis suggested that it has a broader regulatory role in tomato. An evolutionary constraint may underlie this phenomenon, as TF–target interactions involving hub genes are more conserved. Disruptions in these interactions are more likely to be deleterious, leading to reduced genetic diversity and slower evolutionary rates among hub TFs. By contrast, tissue-specific TFs, which are less connected, have been reported to evolve faster (Mack et al., 2019).

### Biological validation of GRNs involved in ripening

We used the fruit-specific GRN to identify important TF regulators of fruit ripening, including *Sl*RIN and TAGL1 (Karlova et al., 2014). To assess the accuracy of our networks, we compared their predicted TF–target interactions against experimentally validated target lists, which included genes confirmed through TF-binding studies (Fujisawa et al., 2013; Zhong et al., 2013; Gao et al., 2019) and regulatory studies of *SlRIN*-deficient plants (Li et al., 2018; Zhao et al., 2018; Gao et al., 2019; Ito et al., 2020). Our results showed significant enrichment between the predicted interactions in our GRNs and these validated targets, supporting the ability of the networks to accurately capture *in vivo* regulatory relationships. Our networks identified *Sl*RIN targets such as *SlFUL1*, previously validated via yeast one-hybrid assays (Fujisawa et al., 2014), as well as essential ripening genes, including *SlACS2*, *SlACS4*, *SlE8*, *SlEXP1*, *SlPSY*, *SlNOR*, and *SlCNR*, which were confirmed using ChIP–PCR (Martel et al., 2011). Similarly, qPCR validation supported TAGL1 targets, including *SlFUL2* (Fujisawa et al., 2014) and ripening-associated genes such as *SlACS2*, *SlETR1*, *SlERF2*, and *SlPL* (Itkin et al., 2009). These findings underscore the predictive strength of our GRNs in identifying key regulatory interactions that govern fruit ripening.

Interestingly, *Sl*RIN and TAGL1 regulate overlapping sets of ripening-related genes, but our fruit-specific GRN does not predict a direct regulatory link between these two TFs. This suggests that their influence on fruit ripening may be mediated through indirect interactions, such as protein–protein interactions or epistatic synergistic control of ripening-responsive genes, as proposed in previous studies (Fujisawa et al., 2014; Jeon et al., 2024). Our GRN identified *Sl*ARF2A and *Sl*ERF2.E2 as central hubs in the fruit-ripening regulatory network, suggesting that these TFs may play crucial roles in modulating the ripening process. *Sl*ARF2A has been implicated in the hormonal regulation of tomato fruit ripening. RNAi-mediated silencing of *SlARF2A* results in ripening defects and ethylene insensitivity, whereas its overexpression (OX) leads to accelerated and uneven ripening (Hao et al., 2015; Breitel et al., 2016). Consistent with these findings, our network analysis identified proposed *Sl*ARF2A targets that aligned with the gene-expression changes in OX-*ARF2A* plants, including *SlETR*, *SlACS4*, *SlAP2A*, *SlETR3*, *SlETR4*, *SlNOR*, and *SlRIN* (Breitel et al., 2016). By contrast, *Sl*ERF.E2 is associated with key ripening regulators and ripening-related genes, as its expression is downregulated in *cnr*, *nor*, and *rin* mutants, but its precise function remains unknown (Liu et al., 2016). Given the potential regulatory roles of these TFs, future studies should use TF-binding assays to confirm their direct interactions with ripening-responsive genes and further characterize their contributions as key hubs in tomato fruit ripening.

### Biological validation of GRNs involved in ABA responses

To further investigate organ-level regulatory mechanisms revealed by our GRNs, we focused on the cellular response to ABA, a biological process consistently enriched across our organ-level gene lists. ABA regulates gene expression primarily through ABA-responsive elements (ABREs), which are recognized by ABF TFs (Uno et al., 2000; Krukowski et al., 2023). Among the ten *ABF* genes described in tomato (Pan et al., 2023), our leaf GRN identified *Sl*ABF2, *Sl*ABF3, and *Sl*ABF5 as central regulators of drought-responsive genes. In *A. thaliana*, between 9 and 13 ABF/AREB family members have been reported (Bensmihen et al., 2002; Fujita et al., 2005); however, only AREB1/*At*ABF2 (AT1G45249), AREB2/*At*ABF4 (AT3G19290), *At*ABF3 (AT4G34000), and *At*ABF1 (AT1G49720) have been functionally validated as important regulators of osmotic and drought stress responses (Kang et al., 2002; Fujita et al., 2005; Yoshida et al., 2010, 2015). These four *At*ABFs exhibit largely overlapping yet cooperative functions, as shown by the enhanced drought sensitivity and ABA resistance observed in triple mutants (Yoshida et al., 2010, 2015). Phylogenetic analyses have demonstrated that homologs of these *At*ABFs are conserved across multiple plant lineages, including bryophytes, lycophytes, monocots, and eudicots, suggesting a conserved role in drought regulation (Fujita et al., 2013; Li et al., 2020b). Our ortholog analysis indicated that *Sl*ABF5 is most closely related to *At*ABF2, whereas *Sl*ABF2 and *Sl*ABF3 are homologous to *At*ABF1 and *At*ABF4, respectively, supporting their roles as conserved regulators of drought stress responses. By contrast, other *Sl*ABFs showed strong differences in their target gene lists, potentially suggesting a divergent function in the regulation of other biological processes. We found that *Sl*ABF6 and *Sl*ABF9 are closely related to *At*ABI5 (At2g36270) and *At*bZIP12/EEL, which are involved in seed maturation and embryo development (Bensmihen et al., 2002; Kim et al., 2002). This regulatory divergence in TF targets underscores the capacity of our GRNs to identify and prioritize functionally relevant TFs within members of the same family, effectively distinguishing regulators of specific regulatory cascades from other TFs potentially involved in other biological processes.

*Sl*ABF2, *Sl*ABF3, and *Sl*ABF5 were found to regulate multiple *PP2C* and *SnRK2* genes, mirroring findings in *Arabidopsis*, in which *At*ABF1, *At*ABF3, and *At*ABF4 bound similar ABA-responsive genes (Song et al., 2016). In functional assays, *SlABF5* overexpression has been shown to enhance drought and salt tolerance in tomato (Orellana et al., 2010). *SlABF2* is also strongly upregulated under drought stress and displays enrichment of the active histone mark H3K4me3, as well as induction in hydrostimulated tomato root tips (Fuentes-Merlos et al., 2023; Wexler et al., 2024). Collectively, these results confirm that our leaf-specific GRN robustly captures core ABA-related gene-regulatory circuits and support the conserved role of *Sl*ABF3 and *Sl*ABF5 in orchestrating drought-responsive gene expression. In addition, our analysis highlights *Sl*ABF2 as a promising, previously uncharacterized regulator of drought responses in tomato.

### *Sl*GBF3 as a novel controller of ABA-related and drought-responsive genes in tomato

Our study identified *Sl*GBF3 as a novel regulatory hub of the ABA-related GRN, suggesting a key role in drought regulation in leaves and potentially in other tomato organs. *Sl*GBF3 is a member of the GBF family, TFs that bind to the G box, a motif commonly found in the promoters of stress-responsive genes (Lu et al., 1996; Sibéril et al., 2001). As found in our analysis, *Sl*GBF3 recognizes a DNA-binding motif similar to that of its *Arabidopsis* homolog, *At*GBF3. *AtGBF3* is reportedly induced by different abiotic stress conditions, including drought, cold, heat, and salt stress, and by biotic stress such as *Pseudomonas* and turnip mosaic virus infection (Dixit et al., 2019). Interestingly, *At*GBF3 contains ABRE motifs in its promoter and has been shown to be induced in response to ABA, consistent with its role in stress defense responses (Lu et al., 1996; Dixit et al., 2019). Moreover, overexpression of *AtGBF3* improves responses to osmotic stress, salinity, and ABA, whereas loss-of-function lines show greater sensitivity to these stresses compared with wild-type plants (Ramegowda et al., 2017). Prior research (Bortolami et al., 2024) linked *SlGBF3* to drought-responsive co-expression modules in tomato, but its regulatory function in stress responses remains unknown. Using our network models, we were able to validate a substantial proportion of ABA-related genes predicted to be *Sl*GBF3 targets as direct binding targets using DAP-seq. Importantly, most of these validated targets were induced by drought, similar to *SlGBF3*, indicating that *Sl*GBF3 may act as a positive controller of its ABA-related targets in this stress response. Our findings suggest that *Sl*GBF3 has a conserved function analogous to that of its *Arabidopsis* homolog, with enrichment of target genes involved in water deprivation, abiotic stimulus response, and response to ABA. In addition to *Arabidopsis*, the function of *Sl*GBF3 homologs in drought responses has also been described in maize, in which knockdown of *ZmGBF3* reduced physiological performance in response to water deprivation (Ramegowda et al., 2017). In addition, overexpression of finger millet *EcGBF3* in *Arabidopsis* improved responses to osmotic stress, salinity, ABA treatment, and drought stress (Ramegowda et al., 2017), indicating a conserved function between dicots and monocots. Interestingly, according to the PLAZA 5.0 database (Van Bel et al., 2022), *SlGBF3* belongs to an orthogroup (ORTHO05D003498) that includes 220 genes from 92 different species, covering different members of the Mesangiospermae and including the basal angiosperm *Amborella trichopoda* and the streptophyte alga *Chara braunii*. This suggests that the function of *Sl*GBF3 is evolutionarily conserved and positions this TF as a potential regulatory hub in different species of agronomic relevance. In addition to the TF itself, several *Sl*GBF3 targets identified in our GRNs have also been suggested as targets for crop improvement. For example, *PP2C phosphatases* have been shown to confer drought tolerance when genetically manipulated in multiple crop species, including rice, soybean, and wheat (Singh et al., 2015; Yu et al., 2019; Zhang et al., 2022; Li et al., 2023). In addition, *SlASR1* (*Solyc04g071610*), a stress-responsive TF directly regulated by *Sl*GBF3 in our network, has been linked to metabolic regulation in tomato, and its orthologs are involved in abiotic stress responses of wheat and maize (Yao et al., 2019; Li et al., 2020a). Together, these findings position *Sl*GBF3 as a promising candidate for targeted genetic manipulation, with potential applications in enhancing responses to stress, including water deprivation, in tomato and other important crops.

#### Exploring tomato GRNs through the TomViz tools

The *S. lycopersicum* organ-level GRNs, available through the rGRN app in the TomViz module of the PlantaeViz platform (Santiago et al., 2024), provide a robust and comprehensive resource for investigating TF–target interactions and organ-level regulatory mechanisms. By integrating extensive datasets and emphasizing regulatory cascades, this tool surpasses traditional GCN-based approaches, which are often limited to fruit tissue and a narrower gene set. The web application offers an accessible yet powerful platform for in-depth regulatory analysis, enabling researchers to explore tomato gene regulation across diverse developmental stages, environmental conditions, and genetic backgrounds. Given the depth and complexity of the data generated in this study, the web interface serves as a critical tool enabling researchers to access, explore, and apply these results for hypothesis generation. Its utility extends to various research contexts, facilitating novel discoveries in tomato biology and advancing functional genomics studies.

## Methods

### Tomato gene annotation update

To track the gene models added in ITAG4.1 and those removed compared with ITAG4.0, an updated version, ITAG4.2-merged, was generated through a conditional merge. Gene models from ITAG4.2 beta (provided by SolGenomics) and ITAG4.0 were integrated into the 4.0 annotation file (.gff3) when their genome coordinates did not overlap with existing entries, resulting in the final ITAG4.2-merged annotation file. To expand the functional annotations for tomato, ITAG4.2-merged protein sequences were analyzed using eggNOG-mapper (Cantalapiedra et al., 2021) to predict GO terms, functional categories, and orthology relationships on the basis of evolutionary genealogy. In addition, functional annotations for all genes in ITAG4.2-merged were generated using default parameters of InterProScan v5.57-90 (Jones et al., 2014) and complemented with information from the PLAZA 5.0 database (Van Bel et al., 2022). The resulting GO annotations for molecular functions and biological processes were consolidated and used to create an updated GO set.

To update the list of TFs in ITAG4.2-merged, we integrated evidence from multiple sources. The selection criteria included gene descriptions from ITAG4.0 and ITAG4.1 (“transcription factor” in the gene name) (Fernandez-Pozo et al., 2015; Hosmani et al., 2019) and the TF catalogs from PlantTFDB (Jin et al., 2017) and ITAK (Zheng et al., 2016). In addition, we added evidence from a keyword search of ITAG4.2-merged GO annotations (“transcription factor,” “DNA-binding”), and we also retrieved proteins annotated as TFs from an InterProScan (Jones et al., 2014) analysis run under default settings. Finally, we added TF evidence if the protein had an ortholog in *A. thaliana* that was annotated as a TF in PlantTFDB (Jin et al., 2017), given the considerable percentage of genes in orthologous groups shared between *Arabidopsis* and tomato (Sato et al., 2012). For this step, we performed an ortholog analysis using OrthoFinder v3.0 (Emms and Kelly, 2019). Genes supported by at least three independent lines of evidence were classified as TFs. Finally, manual curation steps were performed to exclude proteins associated with enzymatic activities, non-transcriptional molecular processes (e.g., DNA replication, repair, splicing, translation), transcriptional regulators other than TFs (e.g., basal transcription factors, RNA polyadenylation factors), and chromatin remodeling complex subunits. We associated PWMs with the TFs by combining information obtained from CisBP v2 (Weirauch et al., 2014) with PWM inference from protein sequences using the JASPAR profile inference tool “infer_profile.py” (Castro-Mondragon et al., 2022).

### Processing of RNA-seq data

To obtain tomato RNA-seq datasets, we queried the NCBI Sequence Read Archive (SRA) database using (“*Solanum lycopersicum*”[Organism] AND ILLUMINA[Platform]) NOT (RIP-Seq[Strategy] OR OTHER[Strategy] OR ChIP-Seq[Source] OR METATRANSCRIPTOMIC[Source] OR Bisulfite-Seq[Strategy] OR GENOMIC[Source] OR METAGENOMIC[Source] OR DNase-Hypersensitivity[Strategy] OR WGS[Strategy] OR ncRNA-Seq[Strategy] OR WCS[Strategy] OR degradome OR miRNA-Seq[Strategy] OR small RNA[Title] OR sRNA[Title]) (Leinonen et al., 2011). The metadata were classified by organ of origin following the protocol in Santiago et al. (2024). The libraries were downloaded using SRAtools (Kans, 2010). Adapters were trimmed, and low-quality reads (average quality *q < 30* and length < 20 bases) were filtered out using fastp v0.20.0 (Chen et al., 2018). Reads were aligned to the SL4.0 *S*. *lycopersicum* genome assembly (Hosmani et al., 2019) using STAR v2.7.3 (Dobin et al., 2013). After mapping, a total of 10 618 mRNA-seq libraries were retained. Gene counts were obtained with FeatureCounts v2.0.0 (Liao et al., 2014) using the ITAG4.2-merged annotation. Total counts were normalized to TPM. Finally, genes with ≥5 TPM in at least 10% of all libraries for each organ were classified as expressed, as described in Huang et al. (2018).

### Processing of ChIP-seq data

The query ((“*Solanum lycopersicum*”[Organism] AND ILLUMINA[Platform]) AND ChIP-Seq[Source]) was used to obtain tomato TF-binding (ChiP-seq) datasets from the NCBI SRA website (Leinonen et al., 2011). ChIP-seq libraries were processed using the methods described by ENCODE (Hitz et al., 2023). In brief, the libraries were downloaded from the NCBI SRA using SRAtools (Kans, 2010). Adapters were trimmed, and low-quality reads (average quality *q* < 30 and length < 20 bases) were filtered using Cutadapt v4.9 (Martin, 2011). Each file was mapped with Bowtie2 v2.54 (Langmead and Salzberg, 2012) to the SL4.0 assembly (Hosmani et al., 2019). Alignment files were sorted and filtered with Samtools v1.21 (Li et al., 2009), and peaks were identified with MACS2 v2.2.9.1 (Zhang et al., 2008). Only libraries with ≥80% mapping efficiency and more than 1000 peaks assigned to annotated genes were retained as high-quality datasets for downstream analysis.

### Processing of ATAC-seq and DNase-seq data

The query ((“*Solanum lycopersicum*”[Organism] AND ILLUMINA[Platform]) AND ATAC-Seq[Source] AND DNAse-Seq[Source]) was used to obtain tomato OCS datasets from the NCBI SRA website (Leinonen et al., 2011). A total of 183 open chromatin libraries (DNase-seq and ATAC-seq) were downloaded using SRAtools (Kans, 2010). Reads were trimmed of adapters, and low-quality reads (average quality *q* < 30 and length < 20 bases) were filtered using Cutadapt v4.9 (Martin, 2011). The ATAC-seq libraries were processed following the protocol described by Reynoso et al. (2019), and the DNase-seq libraries were processed following Moyano et al. (2021). In brief, reads were mapped to the SL4.0 genome assembly (Hosmani et al., 2019) using Bowtie2 v2.54 (Langmead and Salzberg, 2012). The ATAC-seq alignments were sorted and filtered with Samtools v.1.21 (Li et al., 2009), and peaks were identified with HOMER v4.11 (Heinz et al., 2010). DNase-seq regions were mapped into DNase hypersensitive sites using HOTSPOT (Meuleman et al., 2020). OCS peak files were then merged by experiment and converted into FASTA sequences with BedTools v2.31.1 (Quinlan and Hall, 2010).

### Determination of TF-binding sites using FIMO

TF–target datasets were generated by mapping TF DNA-binding preferences, represented as PWMs, to genomic regions of the *S. lycopersicum* SL4.0 genome assembly using the FIMO search tool (Grant et al., 2011). For query sequences, we used all promoter sequences (2 kb upstream of the TSS of each gene) or organ-level OCS sequences. For OCS sequences, the results were assigned to genes using the BedTools command ClosestGene (Quinlan and Hall, 2010).

### GENIE3 inference of regulatory interactions

Raw gene counts for each organ, as well as the updated list of TFs, were provided as input to the GENIE3 algorithm (Huynh-Thu et al., 2010). As previously shown for RNA-seq data, the use of raw expression data does not affect the regulatory inference performance of GENIE3 (Aibar et al., 2017; Escorcia-Rodríguez et al., 2023). The GENIE3 tool was run using the random forest method (default choice), restricting the candidate regulators to the updated list of TFs, with set.seed(123) for reproducibility across runs, K = sqrt (default setting), and 2000 trees (*n*_Trees_ = 2000) to reduce stochastic fluctuations from the random sampling of trees and obtain more stable regulatory edge weights. The output scores were used to create subnetworks based on the top 1%, 2%, 5%, 8%, and 10% of TF–target pairs. These thresholds were consistent with previously published GENIE3 networks (Huang et al., 2018; Cuesta-Astroz et al., 2021; Olivares-Yañez et al., 2021).

### Generation of co-expression networks

To generate co-expression networks for tomato organs, we used the pipeline described by Orduña et al. (2023). In brief, raw data were normalized to fragments per kilobase per million mapped reads (FPKM), and genes with fewer than 0.5 FPKM in every run of the SRA study were discarded. Pearson correlation coefficients (PCCs) were calculated for each gene against all other genes across all individual count matrices and ranked in descending order. Ranked PCC values were used to compute highest reciprocal ranking (HRR) matrices considering only the top 1% best-ranked genes, using the formula HRR(A,B) = max(rank(A,B), rank(B,A)). The GCNs were generated by computing the frequency of co-expression interaction(s) across individual HRR matrices. As a noise-filtering step, only the top 1% frequency values for each gene were retained.

### Evaluation of network performance

To evaluate the performance of the predicted GRNs in capturing experimentally validated regulatory interactions, we followed the protocol of Contreras-López et al. (2022) to compute AUROC and AUPR values. In brief, these analyses were performed using organ-level GRNs and tested against validated regulatory interactions derived from ChIP-seq datasets. Gene interactions were filtered to retain only regulatory genes present in both the GENIE3-inferred and ChIP-seq networks, with edges assigned as binary labels indicating validation status. True- and false-positive rates were calculated using the “precrec” v0.14.4 package in R. To assess statistical significance, AUROC and AUPR values were compared against those of 1000 randomized networks generated by shuffling edge weights. Percentile-based confidence intervals (2.5%–97.5%) were used to benchmark the performance of the GENIE3 network, and significance was determined via permutation testing.

### Visualization of GRNs and network analysis

Network visualizations were generated using Cytoscape v3.10.1 (Shannon et al., 2003), and network topology analyses were performed using the Cytoscape NetworkAnalyzer tool. The R package "Influential" v2.2.9 (Salavaty et al., 2020) was used to identify the TF hubs with the highest IVI.

### Gene set enrichment analysis

GSEA was performed to identify over-represented biological process GO terms using a hypergeometric test with Benjamini–Hochberg FDR correction (threshold < 0.05). The analysis was performed using the BinGO v3.0.5 tool (Maere et al., 2005) within Cytoscape, with input from the updated tomato 4.1c GO term catalog. The REVIGO v.1.8.1 (Supek et al., 2011) web application was used to refine and group GO terms; To focus on more specific biological processes, we selected GO terms; GO terms within levels 5–7.

### Generation of DAP-seq libraries

Genomic DNA (gDNA) was extracted from fully expanded mature leaves of 3-month-old *S.* *lycopersicum* cv. Moneymaker plants using the Wizard Genomic DNA Purification Kit (Promega, Madison, WI, USA), according to the manufacturer’s instructions. gDNA was fragmented to an average size of ∼200 bp using an M220 sonicator (Covaris, Woburn, MA, USA). The fragmented gDNA underwent end repair, A-tailing, and ligation of Illumina adapters. The full-length coding sequence of *SlGBF3* (*Solyc03g120460*) was amplified from cDNA obtained from leaves of *S*. *lycopersicum* cv. Moneymaker using primers SlGBF3_Fw (5′-CAC CAT GGG AAA TAG TGA GGA TGG GAA ATC ATG TAA GC-3′) and SlGBF3_Rv (5′-TCA CCC AGC TGC TAC TGC ATC A-3′). PCR products were cloned into the pFN19K_HaloTag-T7-SP6 Flexi expression vector (Promega), with the HaloTag at the N terminus. Expression of the Halo-tagged fusion protein was performed using the TNT SP6 Coupled Wheat Germ Extract System (Promega). HaloTag-ligand conjugated magnetic beads (Promega) were used to pull down the Halo-tagged TF. Pulled-down TFs were exposed to adapter-ligated gDNA libraries. Bound DNA was eluted, and sequencing libraries were generated by PCR amplification with Illumina TruSeq Universal and Index primers. An empty expression vector was used as a negative control to account for non-specific DNA binding (input library). Libraries were sequenced on an Illumina NovaSeq 6000 platform (150-bp paired end reads, approximately 22 million reads per library). Two replicates were used per experiment.

### Processing of DAP-seq data

Raw sequence data were processed using the pipeline described by Hutin et al. (2023). In brief, adapter sequences were trimmed, and low-quality reads (average quality *q* < 30 and length < 20 bases) were filtered using Cutadapt v4.9 (Martin, 2011). Filtered reads were mapped to the *S. lycopersicum* genome assembly SL4.0 (Hosmani et al., 2019) using Bowtie2 v2.5.4 (Langmead and Salzberg, 2012). Alignment files were sorted, filtered, and deduplicated using Samtools v1.21 (Li et al., 2009). Peaks were identified using MACS2 v2.2.9.1 (Zhang et al., 2008) with the parameters -f BAMPE -q 0.001 --call-summits, using the empty vector control as input to remove background signal and ensure specificity of TF binding. The narrowPeaks from both replicates were merged into consensus peaks using the MSPC package (Jalili et al., 2015) to integrate replicates on the basis of statistical significance. Peaks were annotated to nearby genes using “bedtools window” (v2.30.0) (Quinlan and Hall, 2010) with a window of 2000 bp around the TSSs obtained from the ITAG4.2-merged annotation. To refine the assignment, the midpoint of each peak was calculated, and only peaks located between −2000 bp upstream and +500 bp downstream of a TSS were retained. Finally, TF-binding motifs were identified from the consensus peaks using MEME-ChIP (v5.1.1) (Machanick and Bailey, 2011).

### TomViz-GRNs web platform access and usage

The *S.* *lycopersicum* organ-level GRNs generated in this study are publicly available via the TomViz platform at PlantaeViz (https://plantaeviz.tomsbiolab.com/tomviz) (Santiago et al., 2024) under the “Tomato rGRNs” application. The development and general tools of the PlantaeViz platform have been described previously (Santiago et al., 2024).

## Data and code availability

The organ-level GRNs can be extracted and visualized through the rGRN app in the TomViz module of the PlantaeViz platform (Santiago et al., 2024), available at https://plantaeviz.tomsbiolab.com/tomviz. Supplemental Tables 5, 8, and 11 provide metadata for the public RNA-seq, ATAC-seq, and DNase-seq experiments, as well as the ChIP-seq libraries analyzed in this study. The DAP-seq data generated for this article are available in the NCBI SRA database under accession number SRA: PRJNA1236412. The scripts used to analyze RNA-seq, ATAC-seq, DNase-seq, ChIP-seq, and DAP-seq data, as well as the GENIE3 network generation pipeline and all GRNs, are available at https://github.com/ibioChile/VidalLab/tree/master/Pipelines/Tomato_GRNs_repository. In addition, the repository includes the ITAG4.2-merged GFF annotation, protein FASTA, and transcript FASTA files, which are also provided as Data S2. Genome annotation (ITAG4.2-merged, GFF3 format), Data S3. Protein sequences (ITAG4.2-merged, FASTA), Data S4. Transcript sequences (ITAG4.2-merged, cDNA FASTA).

## Funding

This work was supported by the 10.13039/501100020884Agencia Nacional de Investigación y Desarrollo (ANID)-Millennium Science Initiative Program (Millennium Institute for Integrative Biology iBio ICN17_022 to E.A.V., J.M.A., and J. Canales, and Millennium Nucleus in Data Science for Plant Resilience NCN2024_047 to E.A.V. and J.M.A.); ANID-Fondo de Desarrollo Científico y Tecnológico (FONDECYT) (grants 1211130 and 1250631 to E.A.V., 1230833 and 1211040 to J. Canales, and 1210389 and 1250403 to J.M.A.); ANID-Anillo (grant ACT210007 to E.A.V.); ANID-Vinculación Internacional (grant FOVI230159 to E.A.V., J. Canales, J.M.A., and J.T.M.); Ministerio de Ciencia, Innovación y Universidades (MCIU, Spain) (grant Valinet-PID2021-128865NB-I00 to J.T.M.), 10.13039/501100011033Agencia Estatal de Investigación (AEI, Spain) and 10.13039/501100008530Fondo Europeo de Desarrollo Regional (FEDER, European Union) to J.T.M.; doctoral grant GVA-PROMETEO/2021/056-01 to A.S.; and ANID-Beca Doctoral 21230478 to J.D.F. and 21230939 to D.L.-S. These funding agencies were not involved in the design of the study, data collection and analysis, interpretation of data, or writing of the manuscript.

## Acknowledgments

This research was supported by the computing infrastructure of the Center for Genomics and Bioinformatics, Universidad Mayor, and the HPC cluster Garnatxa at the Institute for Integrative Systems Biology (I^2^SysBio). We thank the staff at SolGenomics (Dr. Surya Saha) for providing the iTAG4.2 beta annotation file. No conflict of interest declared.

## Author contributions

Conceptualization, J.D.F., J. Canales, J.M.A., J.T.M., and E.A.V.; data curation, J.D.F., D.N.-P., and A.S.; investigation, J.D.F., A.C., D.N.-P., and A.S.; formal analysis, J.D.F., D.N.-P., A.S., T.C.M., J. Canan, S.C.-R., D.L.-S., L.M., and N.R.J.; supervision, J. Canales, J.M.A., J.T.M., and E.A.V.; writing – original draft, J.D.F. and E.A.V.; writing – review and editing, J.D.F., D.N.-P., A.S., N.R.J., J. Canales, J.M.A., J.T.M., and E.A.V. All authors provided critical feedback and approved the final version of the manuscript.

## Supplemental information

Data S1. Supplemental Tables 2–9, 11, and 13–20

Data S2. Genome annotation (ITAG4.2-merged, GFF3 format)

Data S3. Protein sequences (ITAG4.2-merged, FASTA)

Data S4. Transcript sequences (ITAG4.2-merged, cDNA FASTA)

Document S2. Article plus supplemental information
