# Supplementary material for: Organ-level gene-regulatory networks inferred from transcriptomic data reveal context-specific regulation and highlight novel regulators of ripening and ABA-mediated responses in tomato
Source: Plant Commun. 2025 Sep 3;6(11):101499. doi: 10.1016/j.xplc.2025.101499 (PMC12785168; doi:10.1016/j.xplc.2025.101499)
Supplement: Document S1. Supplemental Figures 1–13 and Supplemental Tables 1, 10, and 12 [file mmc1.pdf]

Plant Communications, Volume *6*

Supplemental information

Organ-level gene-regulatory networks inferred from transcriptomic data reveal context-specific regulation and highlight novel regulators of ripening and ABA-mediated responses in tomato

José D. Fernández, David Navarro-Payá, Antonio Santiago, Ariel Cerda, Jonathan Canan, Sebastián Contreras-Riquelme, Tomás C. Moyano, Diego Landaeta-Sepúlveda, Lorena Melet, Javier Canales, Nathan R. Johnson, José M. Álvarez, José Tomás Matus, and Elena A. Vidal

Supplemental information

Organ-level GRNs inferred from transcriptomic data reveal context-specific regulation and highlight novel regulators of ripening and ABA-mediated responses in tomato.

José D. Fernández, David Navarro-Payá, Antonio Santiago, Ariel Cerda, Jonathan Canan, Sebastián Contreras-Riquelme, Tomás C. Moyano, Diego Landaeta-Sepulveda, Lorena Melet, Javier Canales, Nathan R. Johnson, José M. Álvarez, José Tomás Matus, Elena A. Vidal.

August 2025

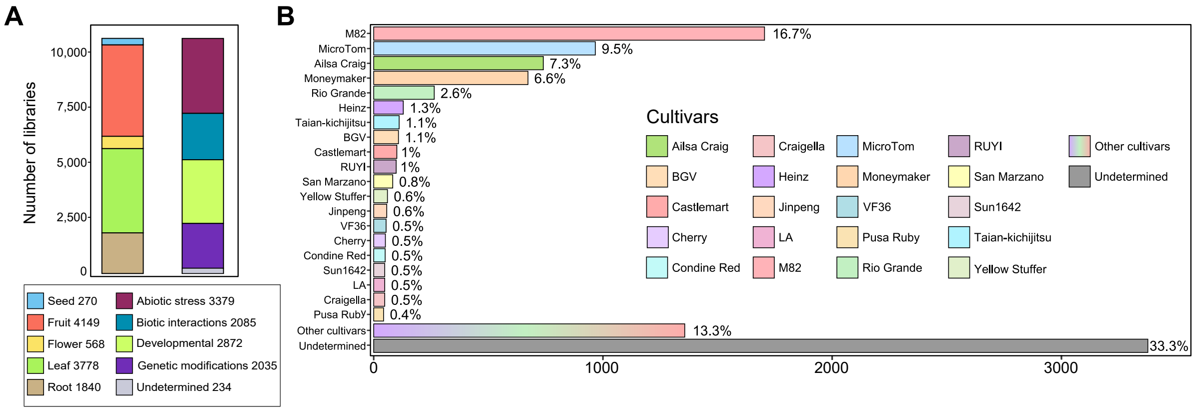

Supplementary Figure S1. Distribution of tomato transcriptomes.

(A) Stacked bar plot showing the number of transcriptomic libraries classified by organ of origin (Seed, Fruit, Flower, Leaf, Root) and by treatment or experimental condition (Abiotic stress, Biotic interactions, Developmental, Genetic modifications, Undetermined). The legend indicates the total number of studies per category.

(B) Horizontal bar plot of the distribution of libraries on the top 20 most abundant tomato cultivars. The percentages next to each bar indicate the relative representation of each cultivar. Cultivars with fewer libraries (<0.4%) are grouped as "Other cultivars;" datasets lacking cultivar annotation are classified as "Undetermined.

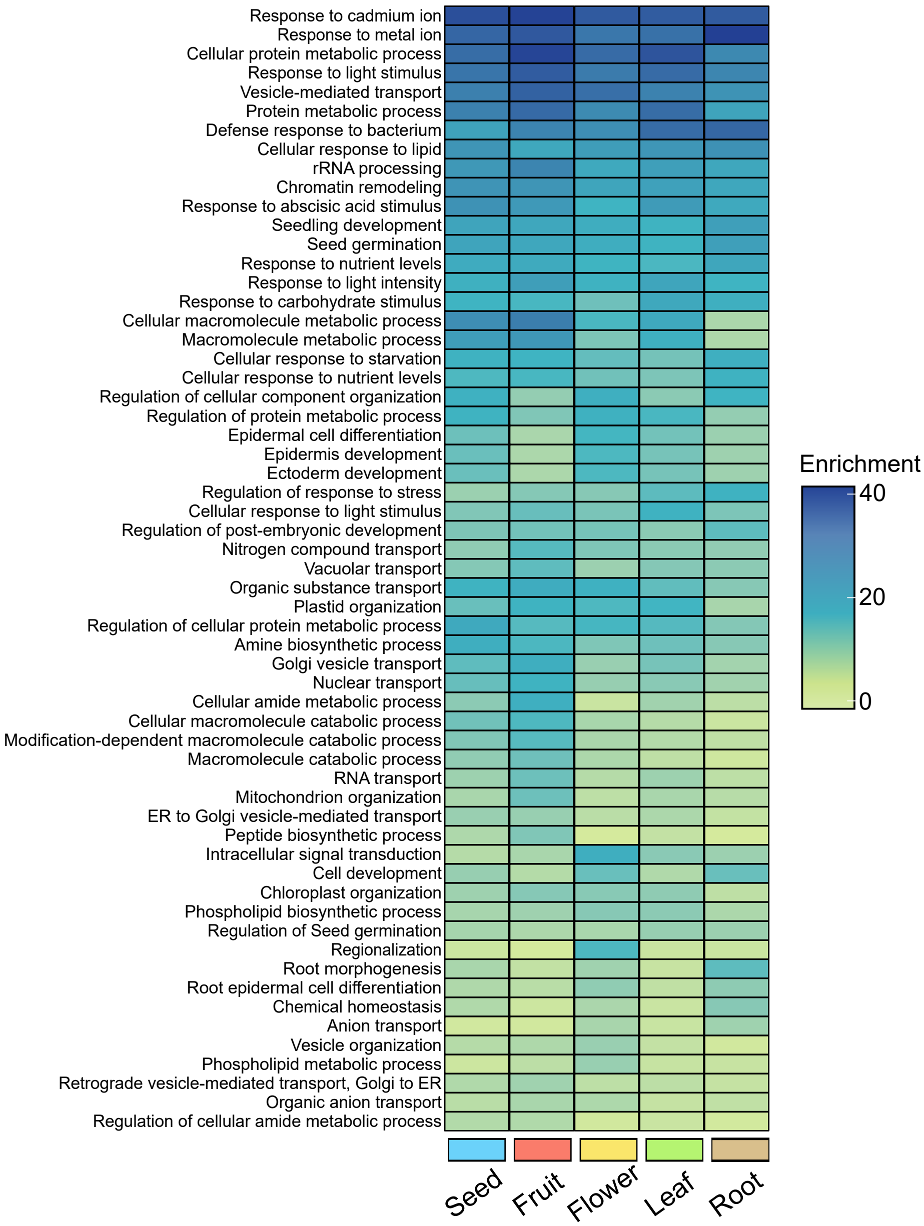

Supplementary Figure S2. Heatmap of Gene Set Enrichment Analysis (GSEA) results for organ-specific gene expression (FDR-adjusted p-value < 0.05).
Color intensity reflects the enrichment values.

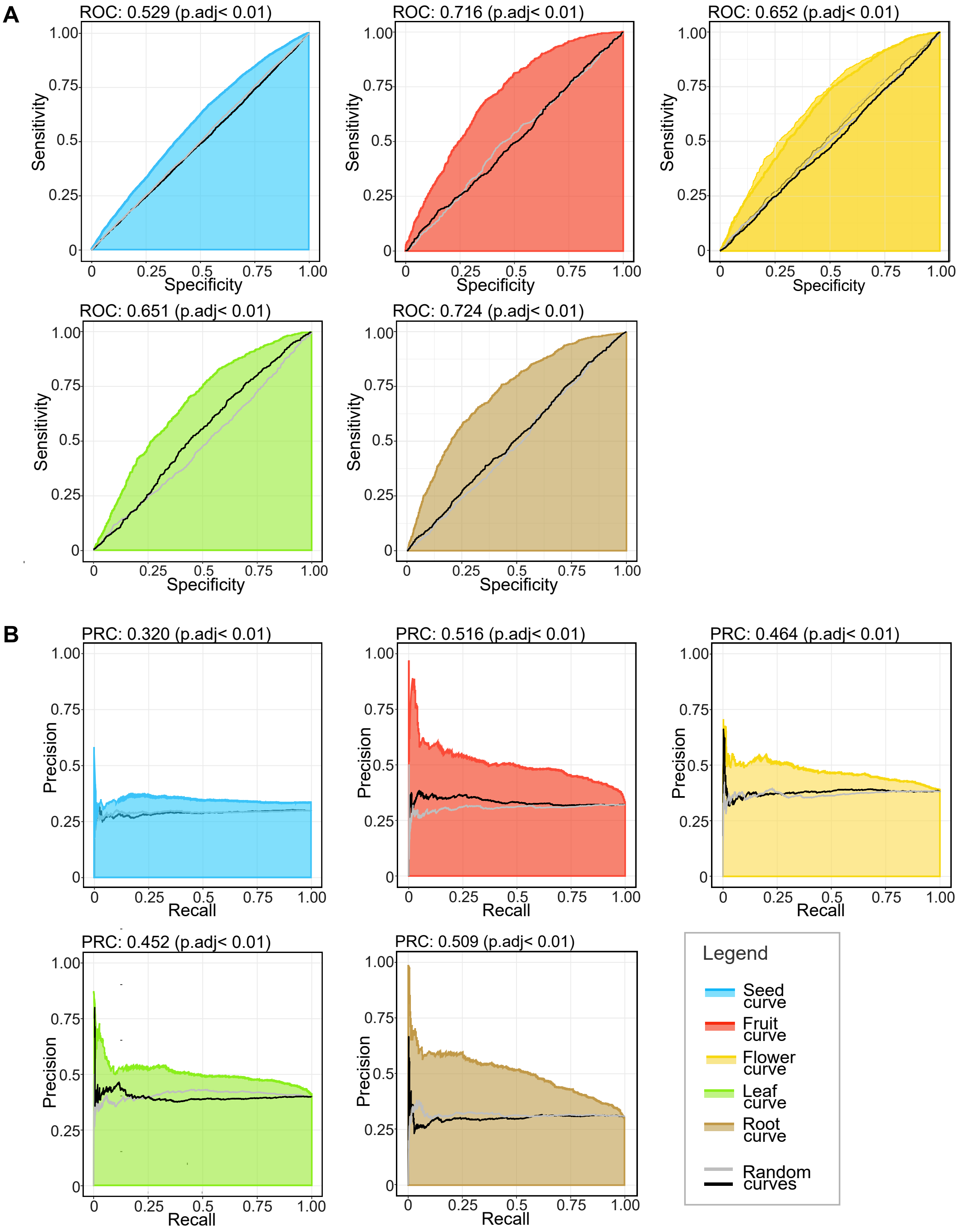

Supplementary Figure S3. Accuracy analysis of organ-specific GRNs.
(A) Receiver Operating Characteristic (ROC) curves and **(B)** Precision-Recall (PR) curves comparing organ-specific GRNs to ChIP-seq validation networks. The shaded areas indicate variability across multiple iterations. Black and grey lines represent the maximum and minimum quartiles of randomly generated TF-target pairings, respectively.

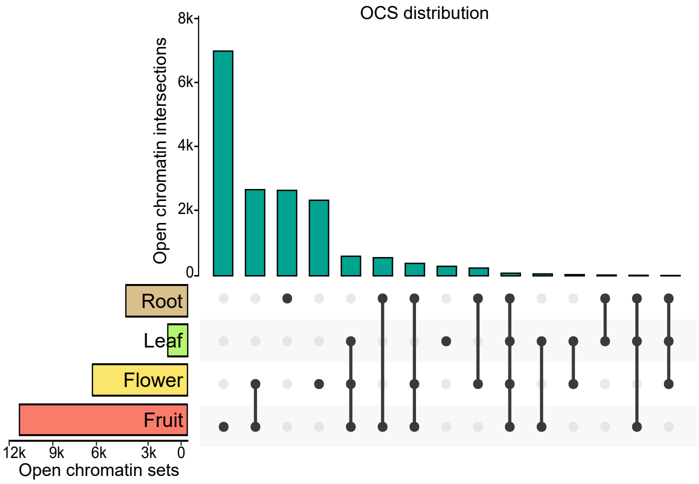

Supplementary Figure S4. Distribution of open chromatin sites (OCSs) across tomato organs. Upsetplot of the distribution of the genomic regions identified as OCS in organ-specific DNAse-seq and ATAC-seq datasets from tomato.

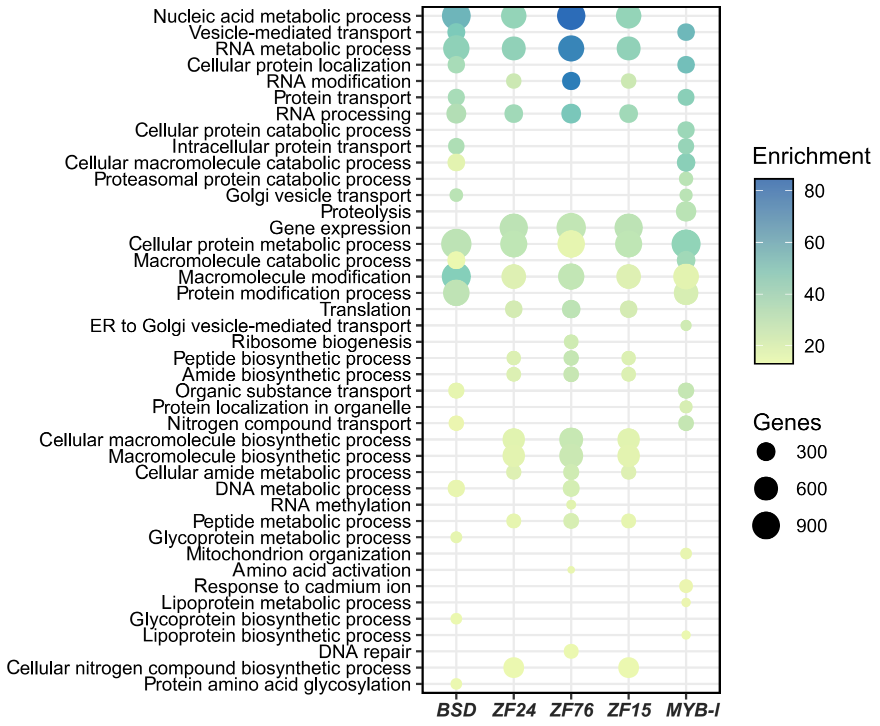

Supplementary Figure S5. GSEA results (FDR-adjusted p-value < 0.05) for the five highest connected TFs shared across organ-level GRNs.
Dot size represents gene number, while color intensity reflects enrichment values per TF.

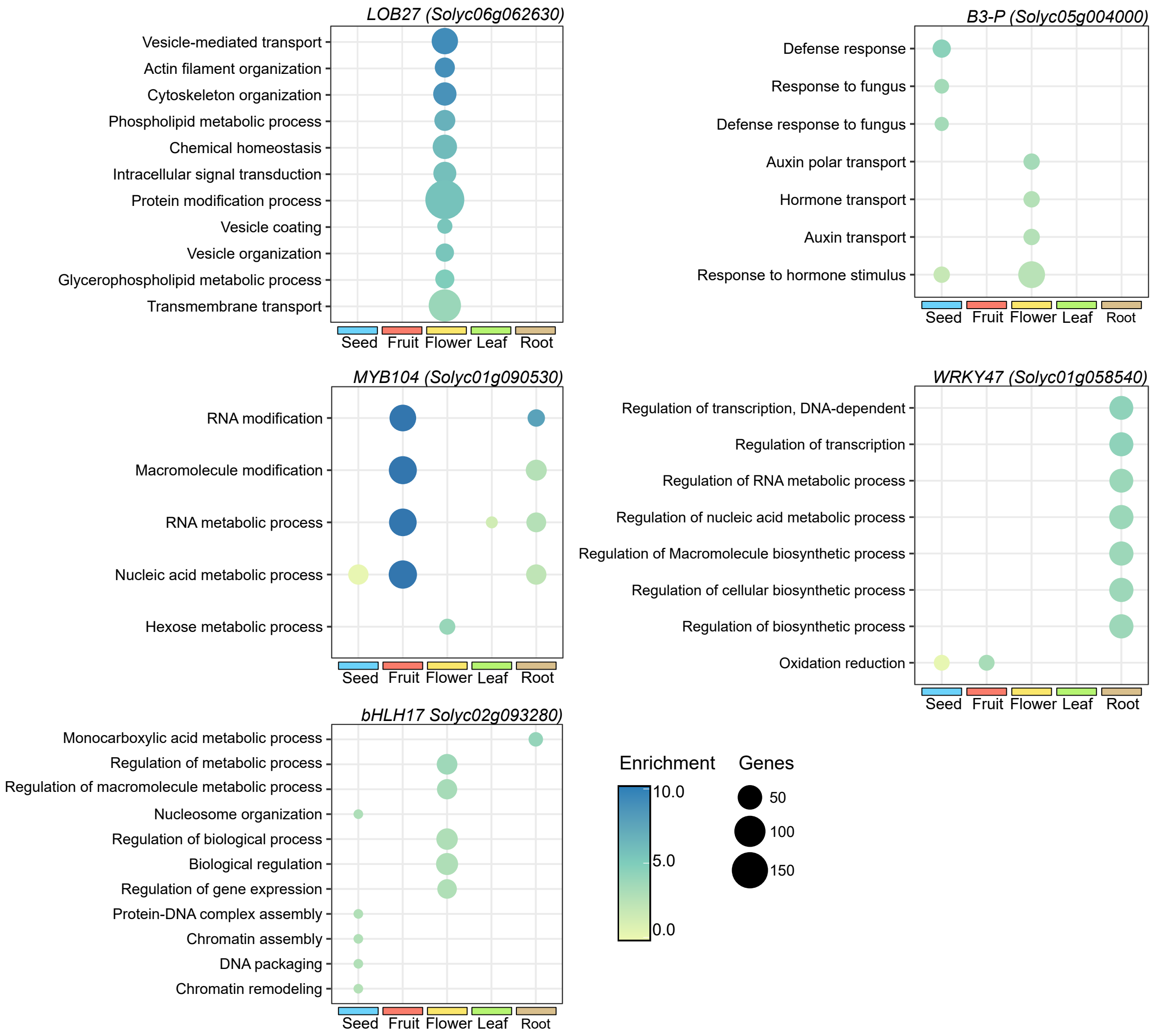

Supplementary Figure S6. GSEA results (FDR-adjusted p-value < 0.05) for the five lowest connected TFs shared across organ-level GRNs.
Dot size represents gene number, while color intensity reflects enrichment values per TF.

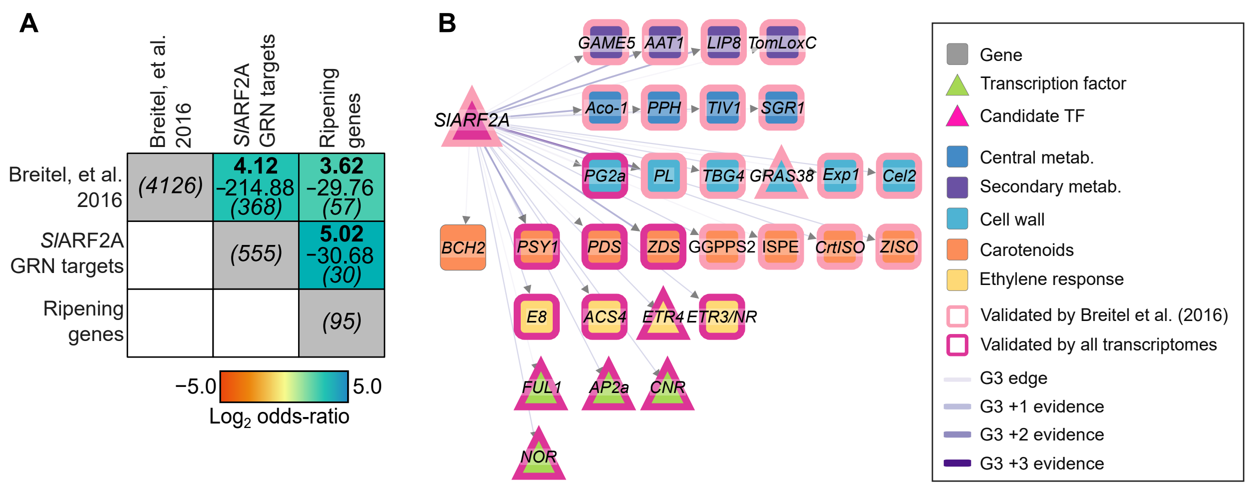

Supplementary Figure S7. Enrichment and network analysis of *Sl*ARF2A targets in tomato fruit GRN.
(A) Box heatmap display enrichment results from a Fisher's exact test ($\log_2$ odds-ratio, $-\log_{10}$ adjusted p-value and intersection size) tested to prior experimental validations (Breitel et al., 2016; Hao et al., 2015) and ripening-associated genes (Li et al., 2019; Zhu et al., 2022).
(B) Subnetwork of ripening-associated gene targets of *Sl*ARF2A. Triangles represent TFs, squares represent target genes. Node colors indicate function, node borders indicate prior experimental validation (Breitel et al., 2016; Hao et al., 2015). Edge darker shades indicate accumulated regulatory evidence.

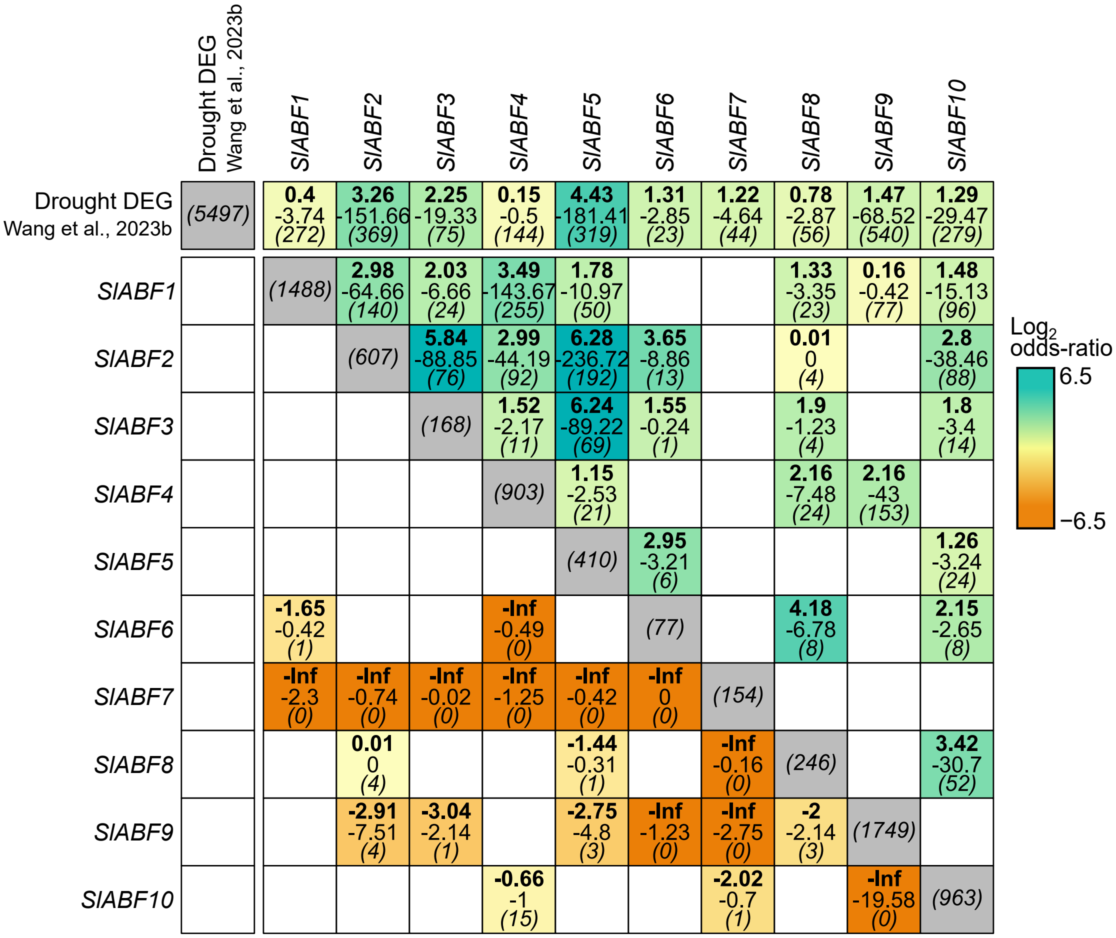

Supplementary Figure S8. Enrichment analysis of *Sl*ABF targets reveal regulatory potential in drought response and functional divergence.

Box heatmaps display enrichment results from a Fisher's exact test ($\log_2$ odds-ratio, $-\log_{10}$ adjusted p-value and intersection size) comparing the predicted targets of *Sl*ABFs (1–10) to the DEGs from drought-stressed leaves (Wang et al., 2023b) as well as pairwise comparisons between the TFs target sets.

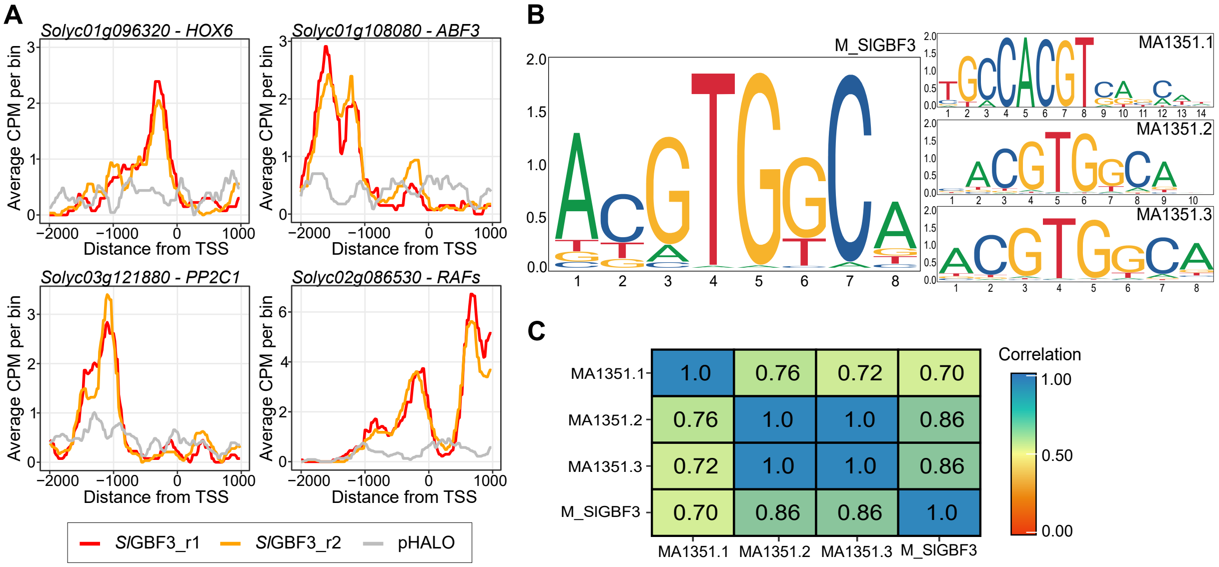

Supplementary Figure S9. Genome-wide identification of *Sl*GBF3 binding targets using DAP-seq.
(A) Line plots of the average DAP-seq signal (CPM per bin) across genomic regions from 2kb upstream to 1kb downstream of the transcription start site (TSS) for selected target genes. Red and orange lines represent biological replicates of *Sl*GBF3 DAP-seq, and the gray line corresponds to the pHALO negative control.
(B) Logo of de novo binding motif identified from *Sl*GBF3 DAP-seq peaks using the MEME suite (Machanick and Bailey, 2011), Right: logos of binding motifs from *At*GBF3 (MA1351.1, MA1351.2, MA1351.3).
(C) Pearson correlation heatmap comparing the enrichment of *Sl*GBF3 binding motif (M_*SlGBF3*) with the *At*GBF3 orthologs.

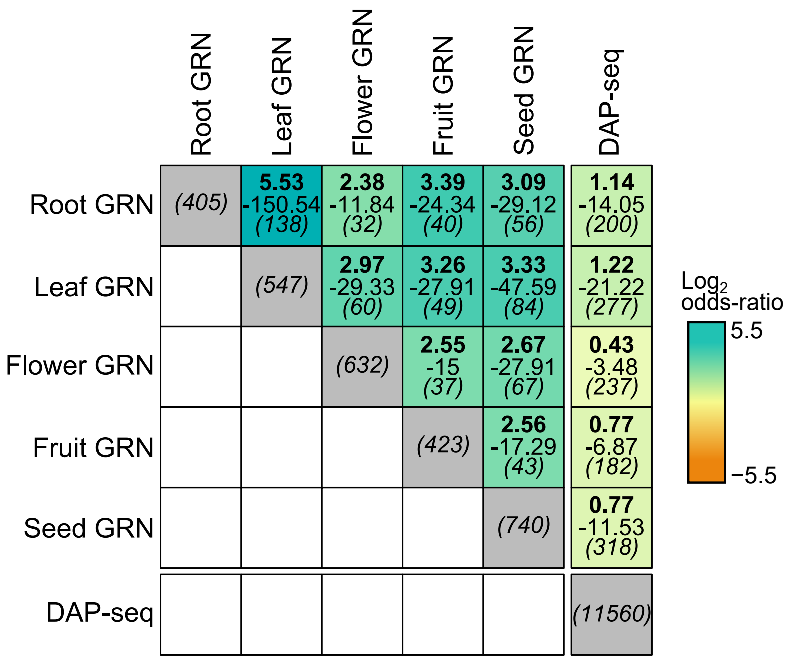

Supplementary Figure S10. Enrichment analysis of *Sl*GBF3 targets in organ-specific GRNs compared to DAP-seq binding targets.

Box heatmap display enrichment results from a Fisher's exact test ($\log_2$ odds-ratio, $-\log_{10}$ adjusted p-value and intersection size).

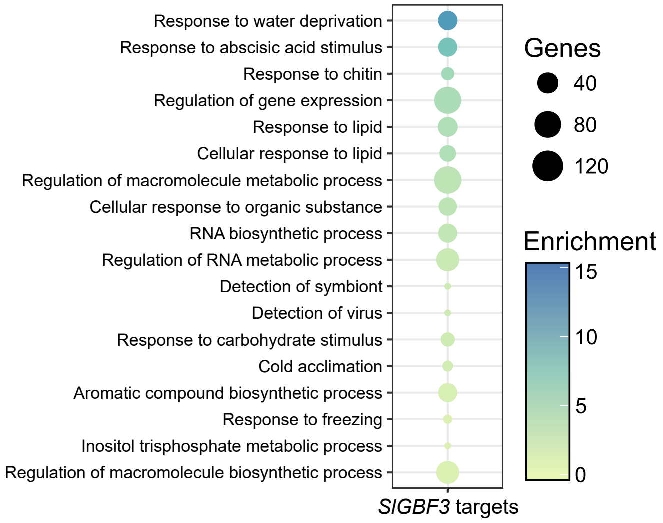

Supplementary Figure S11. GSEA results (FDR-adjusted p-value < 0.05) of *Sl*GBF3 validated targets. Dot size represents gene number, while color intensity reflects enrichment values.

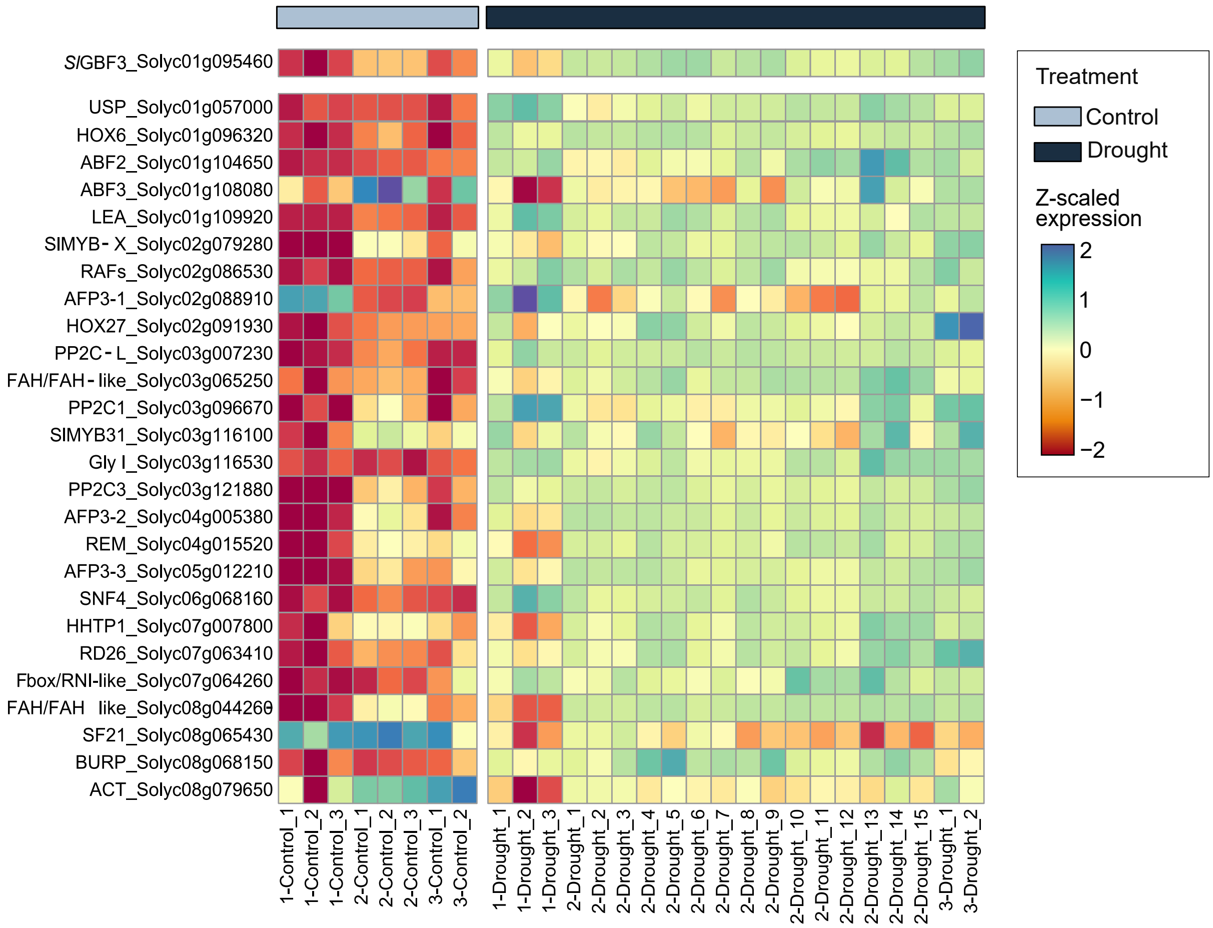

Supplementary Figure S12. Heatmap of expression levels of *Sl*GBF3 and its direct targets across multiple drought responsive transcriptomes.

Gene expression levels (log-transformed and z-scaled) are shown for control libraries (grey) and drought-stressed libraries (dark blue). Genes on the left correspond to *Sl*GBF3 high-confidence targets (HCT) validated by DAP-seq.

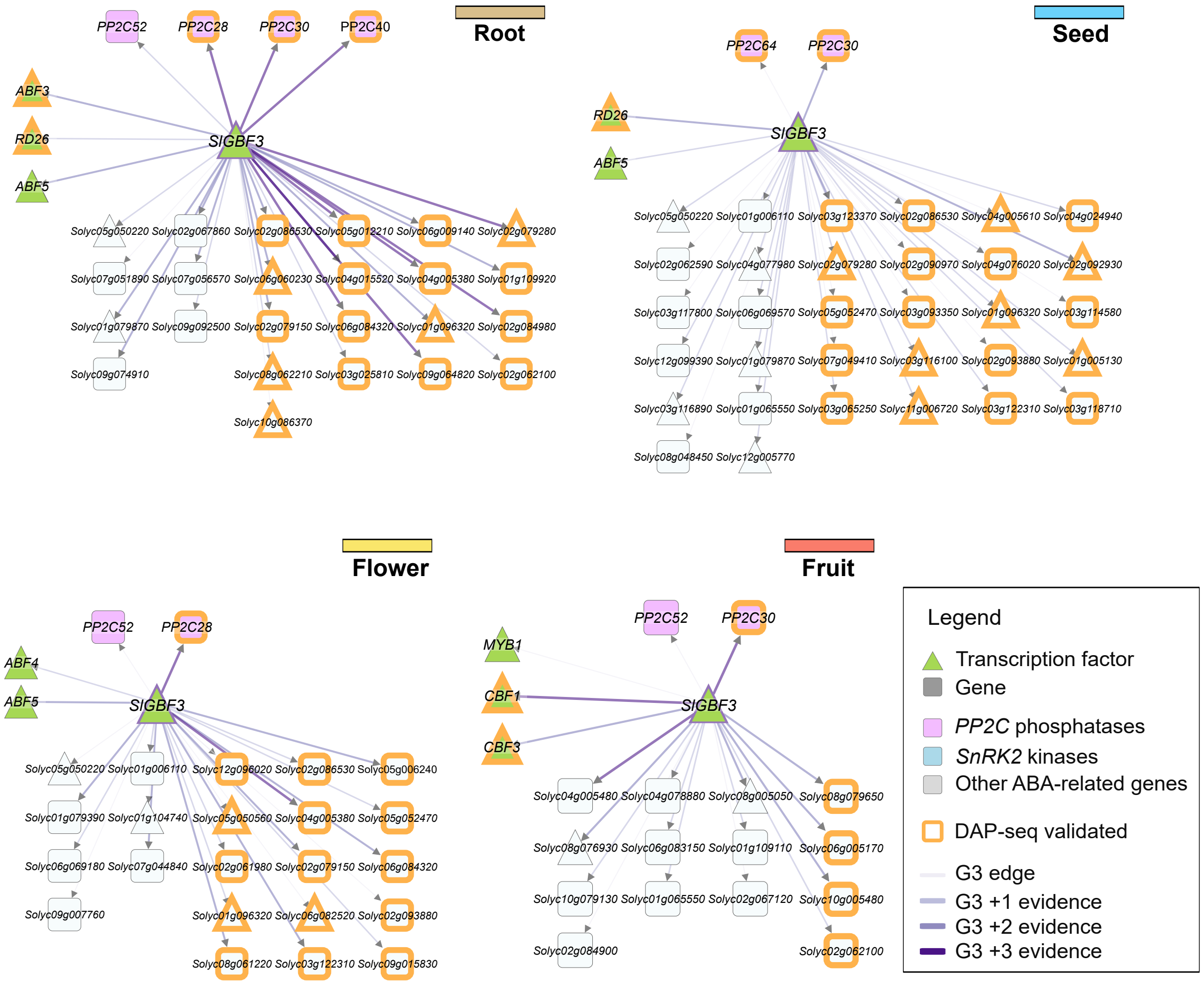

Supplementary Figure S13. Network visualization of *Sl*GBF3-regulated abscisic acid (ABA)-related genes within Root, Seed, Flower and Fruit GRNs.

Triangles represent transcription factors, and squares represent genes. Node colors indicate function: green (important TFs), purple (*PP2C phosphatases*), blue (*SnRK2 kinases*), and grey (other ABA-related genes). Orange-bordered nodes highlight DAP-seq validated genes. Edge color shades represent accumulated regulatory evidence. G3: GENIE3.

Supplementary Table S1. Summary of Tomato Gene Annotations. Overview of total gene counts, transcription factors (TFs), and Gene Ontology (GO) annotations for each tomato genome assembly.

**Source**	**Genome assemby**	**Total genes**	**# TFs**	**Genes with GO annotations**
ITAG2.4	SL2.0	30130	1847	19663
ITAG3.0	SL3.0	34658	1500	-
ITAG4.0	SL4.0	34075	1781	5845
ITAG4.1	SL4.0	34688	2486	13142
ITAG4.2	SL4.0	35932	1832	13544
**ITAG4.2-merged**	**SL4.0**	**37467**	**1840**	**25689**

Supplementary Table S10. Comparative enrichment analysis of GENIE3-predicted TF-target interactions and previously reported networks. Networks used for comparison: PlantRegMap (Regulation merged evidence, Tian et al., 2020), TomatoNet (Full network, Kim et al., 2017). Enrichment results from a Fisher's exact test (log2 fold change, p-value, and intersection size to the ChIP-seq network). -Inf represents $\log_{10}$ adjusted p-values < -400.

Network	PlantTFDB	TomatoNet	GENIE3 2%
Total TFs	308	1,088	1,216
Total Genes	32,925	11,713	22,513
Total edges	325,623	37,267	743,902
log2 Fisher odds ratios	0.75	2.17	3.27
$\log_{10}$ adjusted p-value	-1.08	-16.13	-inf
Number of TF-target pairs in overlap	28	117	2967

Supplementary Table S12. TFs analysis reveals connectivity and target conservation distribution across tomato organs-level GRNs.

Gene	Gene description	TF ID	Organ expression	% Targets consevation	Mean conectivity
Solyc10g005900	BSD domain	BSD	all	57.44	3912.94
Solyc12g017410	Zinc finger transcription factor 76	ZF76	all	50.57	3342.01
Solyc01g110490	Zinc finger transcription factor 15	ZF15	all	44.67	2811.06
Solyc01g079210	Myb/SANT protein	MYB-I	all	48.75	2969.64
Solyc03g026350	Zinc finger transcription factor 24	ZF24	all	52.94	2892.51
Solyc06g062630	LOB domain-containing protein 27	LOB27	all	1.14	63.38
Solyc01g058540	WRKY transcription factor 47	WRKY47	all	7.04	74.96
Solyc01g090530	R2R3MYB transcription factor 104	MYB104	all	3.04	80.33
Solyc05g004000	B3 domain-containing protein	NGA3	all	1.43	83.98
Solyc02g093280	bHLH transcription factor 017	bHLH17	all	3.44	84.87
